# Metabolic regulation of T cell development by Sin1–mTORC2 is mediated by pyruvate kinase M2

**DOI:** 10.1093/jmcb/mjy065

**Published:** 2018-11-14

**Authors:** Xinxing Ouyang, Yuheng Han, Guojun Qu, Man Li, Ningbo Wu, Hongzhi Liu, Omotooke Arojo, Hongxiang Sun, Xiaobo Liu, Dou Liu, Lei Chen, Qiang Zou, Bing Su

**Affiliations:** 1 Shanghai Institute of Immunology, Department of Immunology and Microbiology, Key Laboratory of Cell Differentiation and Apoptosis of Chinese Ministry of Education, Shanghai Jiao Tong University School of Medicine, Shanghai, China; 2 Department of Immunobiology and the Vascular Biology and Therapeutics Program, Yale University School of Medicine, 333 Cedar Street, New Haven, CT, USA

**Keywords:** mTORC2, Sin1, thymocyte development, PPAR-γ, PKM2, metabolism

## Abstract

Glucose metabolism plays a key role in thymocyte development. The mammalian target of rapamycin complex 2 (mTORC2) is a critical regulator of cell growth and metabolism, but its role in early thymocyte development and metabolism has not been fully studied. We show here that genetic ablation of Sin1, an essential component of mTORC2, in T lineage cells results in severely impaired thymocyte development at the CD4^−^CD8^−^ double negative (DN) stages but not at the CD4^+^CD8^+^ double positive (DP) or later stages. Notably, Sin1-deficient DN thymocytes show markedly reduced proliferation and glycolysis. Importantly, we discover that the M2 isoform of pyruvate kinase (PKM2) is a novel and crucial Sin1 effector in promoting DN thymocyte development and metabolism. At the molecular level, we show that Sin1–mTORC2 controls PKM2 expression through an AKT-dependent PPAR-γ nuclear translocation. Together, our study unravels a novel mTORC2−PPAR-γ−PKM2 pathway in immune-metabolic regulation of early thymocyte development.

## Introduction

T cell development depends primarily on glucose metabolism and glycolysis has been shown to play a vital role in the DN3–DN4 transition (Brand and Hermfisse, 1997; Sandy et al., 2012; Buck et al., 2015). Specific extracellular signals have been shown to participate in the regulation of intracellular glucose metabolism during thymocyte development and the PI3K/AKT signaling cascade has been shown to be one of the most important regulators for the glycolytic metabolism to trigger early T cell development (Powell and Delgoffe, 2010; Zeng and Chi, 2013).

The PI3K/AKT signaling cascade mediates cellular metabolism and growth through at least two well defined but distinct mammalian target of rapamycin (mTOR) complexes, mTORC1 and mTORC2, to control T cell development, activation and differentiation (Hung et al., 2012; Cornu et al., 2013; Linke et al., 2017). mTORC1, which consists of its core components Raptor, mLST8, and mTOR, senses cellular nutrients for protein translation and ribosomal biogenesis through phosphorylation of the two critical substrates S6K and 4E-BP1. mTORC2, which consists of its core components Rictor, Sin1, mLST8, and mTOR, is activated by a large spectrum of mitogenic growth factors, cellular stresses, and developmental cues to phosphorylate and regulate a family of protein kinases called AGC family kinases including AKT, PKC, and SGK. AKT is the most well-characterized substrate of mTORC2 and its two critical regulatory phosphorylation sites, Thr450 and Ser473, are stringently regulated by mTORC2 in most cell types including T cells (Jacinto et al., 2006; Facchinetti et al., 2008; Lazorchak et al., 2010; Wu et al., 2011). Phosphorylation of AKT at Thr450 and Ser473 not only controls the expression levels and optimal activation of AKT but also defines the substrate specificity and the resolving phase of AKT activity (Jacinto et al., 2006; Yang et al., 2006; Facchinetti et al., 2008; Su and Jacinto, 2011).

Sin1 is one of the essential core components of mTORC2 and Sin1 deficiency leads to severely impaired phosphorylation of AKT at Ser473 and Thr450 residues due to the disruption of the multi-protein complex of mTORC2 (Jacinto et al., 2006; Liu et al., 2013). T cell development in *Sin1*^−/−^ fetal liver hematopoietic stem cell (FL-HSC) reconstituted mice showed increased nTreg cells as well as increased DN cells as compared to that of wild-type (WT) control FL-HSC reconstituted mice (Chang et al., 2012). Interestingly, in the same study using FL-HSC to co-culture with OP9-DL1 stromal cells in the presence of IL-7, Sin1^−/−^ and WT FL-HSC gave rise to similar pattern of the development of thymocytes, suggesting that the precise *in vivo* role of Sin1 and mTORC2 in T cell development is more complex. The studies focusing on Rictor, another essential component of mTORC2, showed that the Rictor-deficient mice generated by dLck-iCre did not affect normal thymocyte numbers and overall subset population distribution(Lee et al., 2010). In contrast, other studies supported a critical role of mTORC2 in thymocyte development *in vivo* and *in vitro* (Lee et al., 2012; Tang et al., 2012; Chou et al., 2014). Collectively, these studies suggest a very complex regulatory mechanism of mTORC2-dependent thymocyte development.

To investigate the *in vivo* roles of Sin1 in a tissue-specific manner, our laboratory generated *Sin1* floxed mice, which should allow us to study the *in vivo* role of Sin1 in more details with tissue-specific deletion of Sin1. Using this newly established system, we discover a previously unappreciated function of Sin1 in regulating early thymocyte development. This study also leads to the identification of PKM2 as a novel Sin1 substrate to facilitate the mTORC2 function in promoting early T cell development and metabolism.

## Results

### Sin1 plays a cell-intrinsic role in early thymocyte development

**Figure 1:**
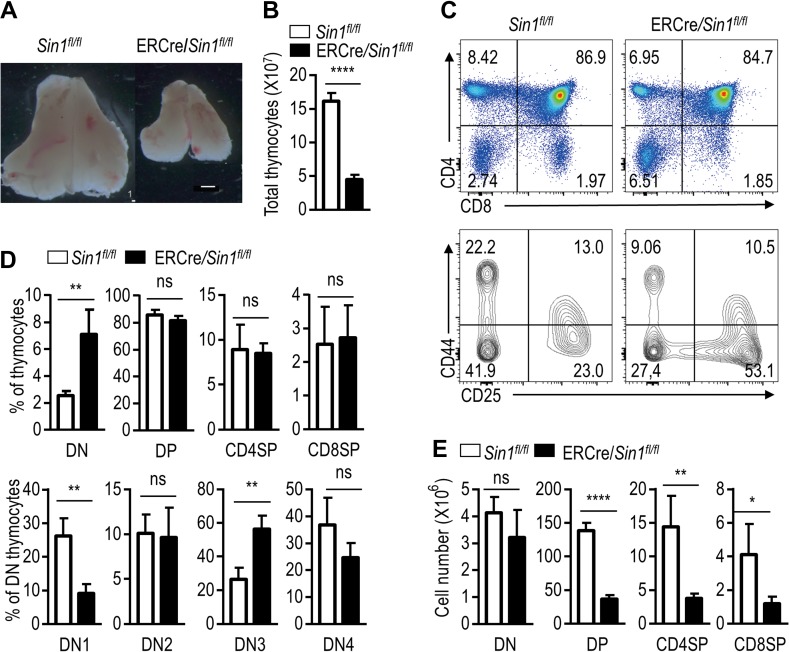
Sin1 deficiency impairs T cell development. (**A**) The size of thymus from tamoxifen-treated *Sin1*^*fl/fl*^ mice and ERCre*/Sin1*^*fl/fl*^ mice. Freshly isolated thymuses were pictured by a stereo microscope. Scale bar, 1 mm. (**B**) The bar graphs represent the number of thymocytes described in **A**. (**C**) Surface staining of total thymocytes from tamoxifen-treated *Sin1*^*fl/fl*^ mice and ERCre*/Sin1*^*fl/fl*^ mice with indicated antibodies. Upper panels are CD4 and CD8 staining. Lower panels are gated on the CD4 and CD8 double negative (DN) subset for further CD44 and CD25 expression analyses. Numbers in the panels show the relative percentage of cells in that area. (**D**) Quantification of thymocyte subsets based on FACS results in **C**. (**E**) The bar graphs illustrate the cell number of different thymocyte subsets. The data shown were calculated from the data in **B** and **D**. Error bars show mean ± SD, *n* = 5. Significance was determined by two-tailed Student’s *t*-test (*P < 0.05; **P < 0.01; ****P < 0.0001; ns, no significant difference).

We recently established a *Sin1* floxed (*Sin1*^*fl/fl*^) mouse line by flanking the exon 4 of *Sin1* with two loxP sites (Materials and Methods; Supplementary Figure S1A). We first analyzed inducible *Sin1*-knockout (named Sin1-iKO) mice when *Sin1* was inducibly deleted by tamoxifen (TM) treatment at an age of 6–8 weeks after crossing *Sin1*^*fl/fl*^ mice to a ROSA26-Cre-ER^T2^ transgenic mouse line to generate ROSA26-Cre-ER^T2^/*Sin1*^*fl/fl*^ (referred to as ERCre/*Sin1*^*fl/fl*^) mice. Sin1 expression in thymocytes and splenocytes was efficiently deleted after TM-treatment (Supplementary Figure S1C and D). Overall, Sin1 deletion had very little effect on the appearance and growth of the Sin1-iKO mice compared with WT mice. However, we found that the inducible Sin1 deletion markedly reduced thymic size and total thymocyte numbers (Figure 1A and B). These data suggest that Sin1 may play a critical role than previously thought during the thymocyte development.

To investigate this, Sin1-deficiency caused thymocyte development defect further, the ratios and total numbers of CD4 and CD8 DN, double positive (DP), and single positive (SP) thymocyte subsets were analyzed. We found that in Sin1-deficient mice, the percentage of DN thymocytes was markedly increased (Figure 1C and D). When DN thymocytes were further analyzed according to their CD25 and CD44 expression, we found that the proportions of DN1 (CD44^+^CD25^−^), DN2 (CD44^+^CD25^+^), DN3 (CD44^−^CD25^+^), and DN4 (CD44^−^CD25^−^) cells were altered as compared to those of WT DN cells (Figure 1C and D). The DN1 cells in *Sin1* KO mice were reduced whereas the DN3 were increased as compared to that of WT DN cells (Figure 1D). Although the relative ratios of DP and SP thymocytes were not significantly changed, their total numbers were decreased significantly in *Sin1* KO mice (Figure 1E).

Thymic stromal cells are important for thymocyte development (Anderson and Takahama, 2012; Cowan et al., 2013). To examine whether Sin1 has a potential role in thymic stromal cells, WT bone marrow (BM) cells from C57BL/6 CD45.1 (B6.SJL-Ptprc^a^ Pepc^b^/BoyJ) mice were adoptively transferred into lethally irradiated *Sin1* KO mice or their littermate WT controls (CD45.2^+^). The BM-reconstituted mice were analyzed 2 months later and the thymocytes developed similarly in these reconstituted mice in terms of the numbers and overall populations (Supplementary Figure S1E and F), suggesting that Sin1 may not be required for thymic stromal cell function in thymocytes development.

To determine if the regulation of thymocyte development *in vivo* by Sin1 is cell intrinsic, BM cells from *Sin1* KO mice or their WT littermate controls (CD45.2^+^) mice were adoptively transferred to lethally irradiated WT C57BL/6 CD45.1 mice and the recipient mice analyzed two months later after reconstitution. The proportions of DN thymocytes and their subsets in *Sin1* KO BM-reconstituted C57BL/6 CD45.1 mice were increased as compared to those in WT BM-reconstituted C57BL/6 CD45.1 mice (Supplementary Figure S2A and B). Furthermore, we used a mixed adoptive transfer model by mixing *Sin1* KO or WT BM cells (CD45.2^+^) with another WT BM cells from C57BL/6 CD45.1 mouse (CD45.1^+^) at roughly 1:1 ratio, respectively, and subsequently transplanted into lethally irradiated CD45.1^+^CD45.2^+^ mice (also C57BL/6 background). The proportions and numbers of DN, DP, and SP thymocytes originated from CD45.1^+^ WT or CD45.2^+^ WT BM cells were similar (Supplementary Figure S3C and D). In contrast, the proportions of DN1–DN4 cells originated from the CD45.2^+^*Sin1* KO BM cells were similarly altered as found in Sin1-iKO mice, and the relative numbers of *Sin1* KO thymocytes were also markedly reduced as compared to the WT control thymocytes in the same mice (Supplementary Figure S3E and F). These data together strongly suggest that Sin1 plays a cell-intrinsic role in thymocyte development *in vivo*.

### Sin1 regulates early thymocyte development

**Figure 2:**
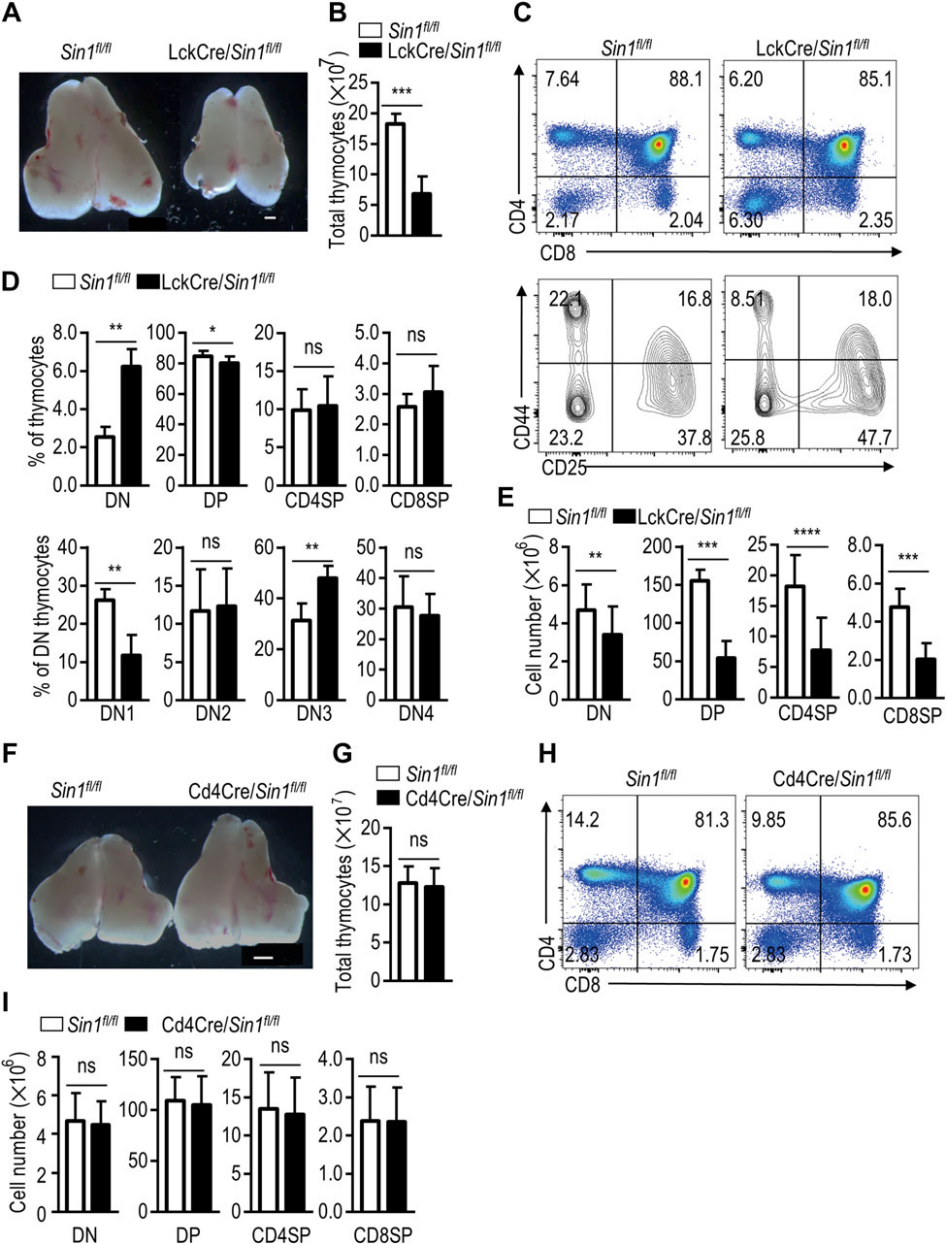
Sin1 is essential for early thymocyte development. (**A**) The size of thymus from *Sin1*^*fl/fl*^ mice and LckCre/*Sin1*^*fl/fl*^ mice. Freshly isolated thymuses were dissected and pictured as described above. Scale bar, 1 mm. (**B**) The summary graphs show the number of thymocytes in different subsets (*n* = 10). (**C**) Surface staining of total thymocytes from *Sin1*^*fl/fl*^ mice and LckCre/*Sin1*^*fl/fl*^ mice with indicated antibodies. Upper panels are CD4 and CD8 staining. Lower panels are gated on the CD4 and CD8 DN subset for further CD44 and CD25 expression analysis. Numbers in the panels show the relative percentage of cells in that area. (**D**) Statistics of thymocyte distribution determined by FACS in **C** (*n* = 10). (**E**) Bar graphs of thymocyte subset cell number (*n* = 10). The data shown were calculated based on **B** and **D**. (**F**) The sizes of thymus from *Sin1*^*fl/fl*^ mice and Cd4Cre/*Sin1*^*fl/fl*^ mice. Freshly isolated thymuses were detected as described above. Scale bar, 1 mm. (**G**) The summary graph showed the number of thymocytes (*n* = 6). (**H**) Surface staining of thymocytes shown in **F**. Total thymocytes are shown based on CD4 and CD8 expression. The numbers in the outlined areas indicate the percentage of cells in each area. (**I**) The quantification of the number of thymocyte subsets based on **G** and **H** (*n* = 6). Error bars show mean ± SD. Significance was determined by two-tailed Student’s *t-*test (*P < 0.05; **P < 0.01; ***P < 0.001; ****P < 0.0001; ns, no significant difference).

To further confirm the cell-intrinsic role of Sin1 in T cell development *in vivo*, LckCre and Cd4Cre transgenic mice were bred with *Sin1*^*fl/fl*^ mice. LckCre and Cd4Cre recombinases are expressed under promoters of the T cell-specific *Lck gene*, which begins expression at the DN2 stage, and *Cd4* gene, which begins expression at later DN4 stage. Specific deletion of Sin1 in T cells was confirmed in LckCre/*Sin1*^*fl/fl*^ mice and Cd4Cre/*Sin1*^*fl/fl*^ mice (Supplementary Figure S4A and B). With no surprise, LckCre/*Sin1*^*fl/fl*^ mice displayed a smaller thymic size, increased DN percentage, altered DN subsets, and reduced total thymocyte number as shown in above experiments done in Sin1-iKO mice, and BM cell reconstitution experiments (Figure 2A–E). The consistent increase in the percentage of DN3 cells (Figure 2C and D) suggested that Sin1 may be involved in transition from the DN3 to DN4 stage through a mTORC2-dependent mechanism since a similar phenotype was observed in Rictor-deficient mice (Tang et al., 2012; Chou et al., 2014).

Surprisingly, we found no obvious defect in thymocyte development in the Cd4Cre/*Sin1*^*fl/fl*^ mice, both the percentages of subsets of DN, DP, and SP cells as well as the total numbers of thymocytes (Figure 2F–I). These results further support our findings that Sin1 is critical for the early DN thymocyte development, and also suggest that Sin1’s function may be dispensable for late-stage thymocyte development.

### Sin1 promotes DN thymocyte proliferation

**Figure 3:**
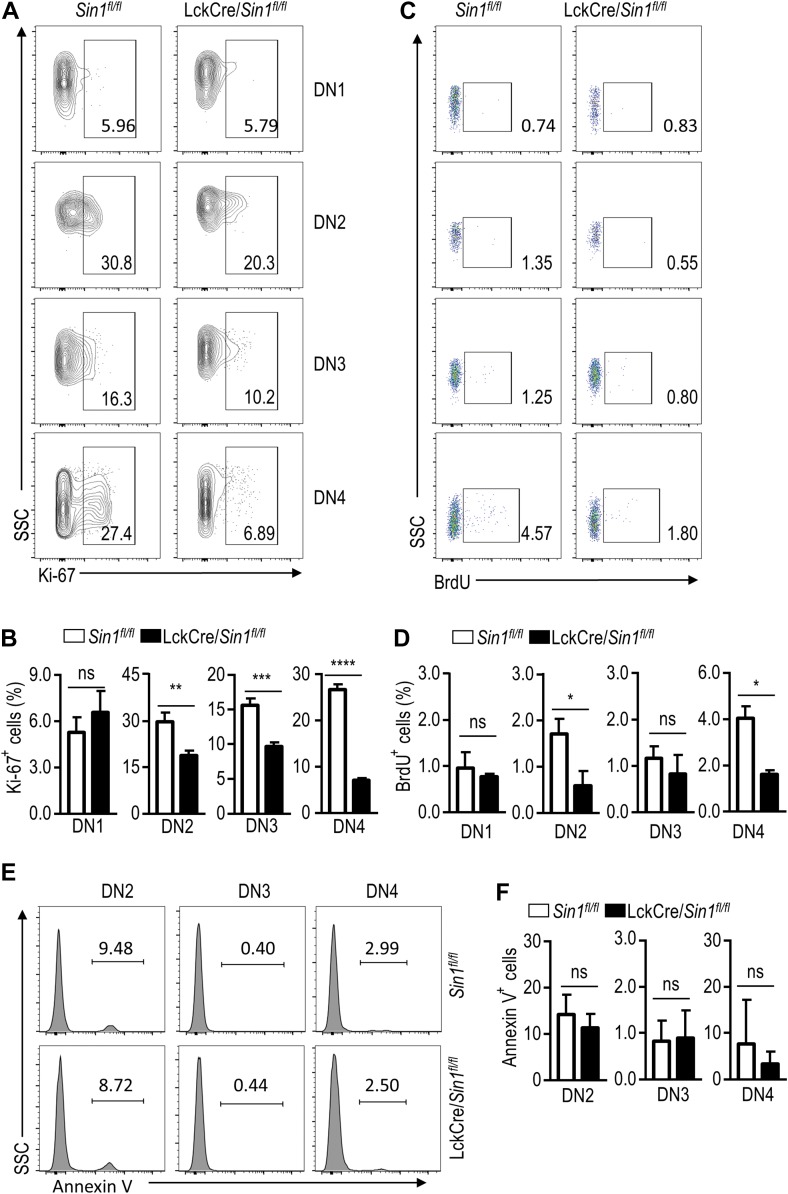
Sin1 maintains thymic cell numbers by promoting cell proliferation. (**A**) Ki-67 staining of DN thymocytes from *Sin1*^*fl/fl*^ mice and LckCre/*Sin1*^*fl/fl*^ mice. The Ki-67-positive positive cells were shown in thymocyte subsets, which were distinguished by surface makers as described above. The numbers in the outlined areas indicate the percentage of cells in each area. (**B**) The proliferation rate of DN thymocytes based on FACS, as described above in **A** (*n* = 3). (**C**) BrdU staining of DN thymocytes from *Sin1*^*fl/fl*^ mice and LckCre/*Sin1*^*fl/fl*^ mice. The mice were sacrificed 2 h after i.p. injection of BrdU. The BrdU-positive cells were shown in thymocyte subsets as described above. The numbers in the outlined areas indicate the percentage of cells in each area. (**D**) Proliferation rate of DN thymocytes based on FACS, as described above in **C** (*n* = 3). (**E**) Annexin V staining of DN thymocytes from *Sin1*^*fl/fl*^ mice and LckCre/*Sin1*^*fl/fl*^ mice. The Annexin V-positive cells were shown in thymocyte subsets as described above. The numbers in the outlined areas indicate the percentage of cells in each area. (**F**) The apoptosis rate of DN thymocytes based on FACS, as described above in **E** (*n* = 3). Error bars show mean ± SD. Significance was determined by two-tailed Student’s *t*-test (*P < 0.05; **P < 0.01; ***P < 0.001; ****P < 0.0001; ns, no significant difference).

Because the decreased thymocyte number could be due to increased apoptosis or reduced cell proliferation, we next examined the apoptosis and proliferation of DN thymocytes from LckCre/*Sin1*^*fl/fl*^ mice and their WT littermate controls. Freshly isolated thymocytes were stained with an antibody against nuclear protein Ki-67 to reveal the proliferative capacity of each subset of DN thymocytes. The percentages of Ki-67-positive cells from LckCre/*Sin1*^*fl/fl*^ mice were markedly reduced at the stages of DN2, DN3, and DN4 compared with those from their WT littermate controls (Figure 3A and B). Additionally, we also labeled LckCre/*Sin1*^*fl/fl*^ and their WT littermate control mice with BrdU *in vivo*, and the BrdU-positive cells were analyzed from different subsets of DN thymocytes. These experiments showed again that the DN2–DN4 cells from the LckCre/*Sin1*^*fl/fl*^ mice had markedly reduced BrdU-positive cells as compared to those from control mice (Figure 3C and D). However, DN thymocyte apoptosis was not much different between the LckCre/*Sin1*^*fl/fl*^ and WT control mice (Figure 3E and F). Finally, we examined the expression of TCRβ and CD127 in *Sin1* KO and WT thymocytes, but found no differences (Supplementary Figure S5). Together, these data suggest that *Sin1* is crucial for proper DN thymocyte proliferation. Although this conclusion is consistent with the previous findings showing that Rictor and mTORC2 regulate thymocyte development (Tang et al., 2012), it may have very different mechanisms since we did not observe altered thymocyte CD127 and TCRβ expression in Sin1-deficient mice (Chou et al., 2014).

### Sin1 is required for glycolysis and oxidative responses in developing thymocytes

**Figure 4:**
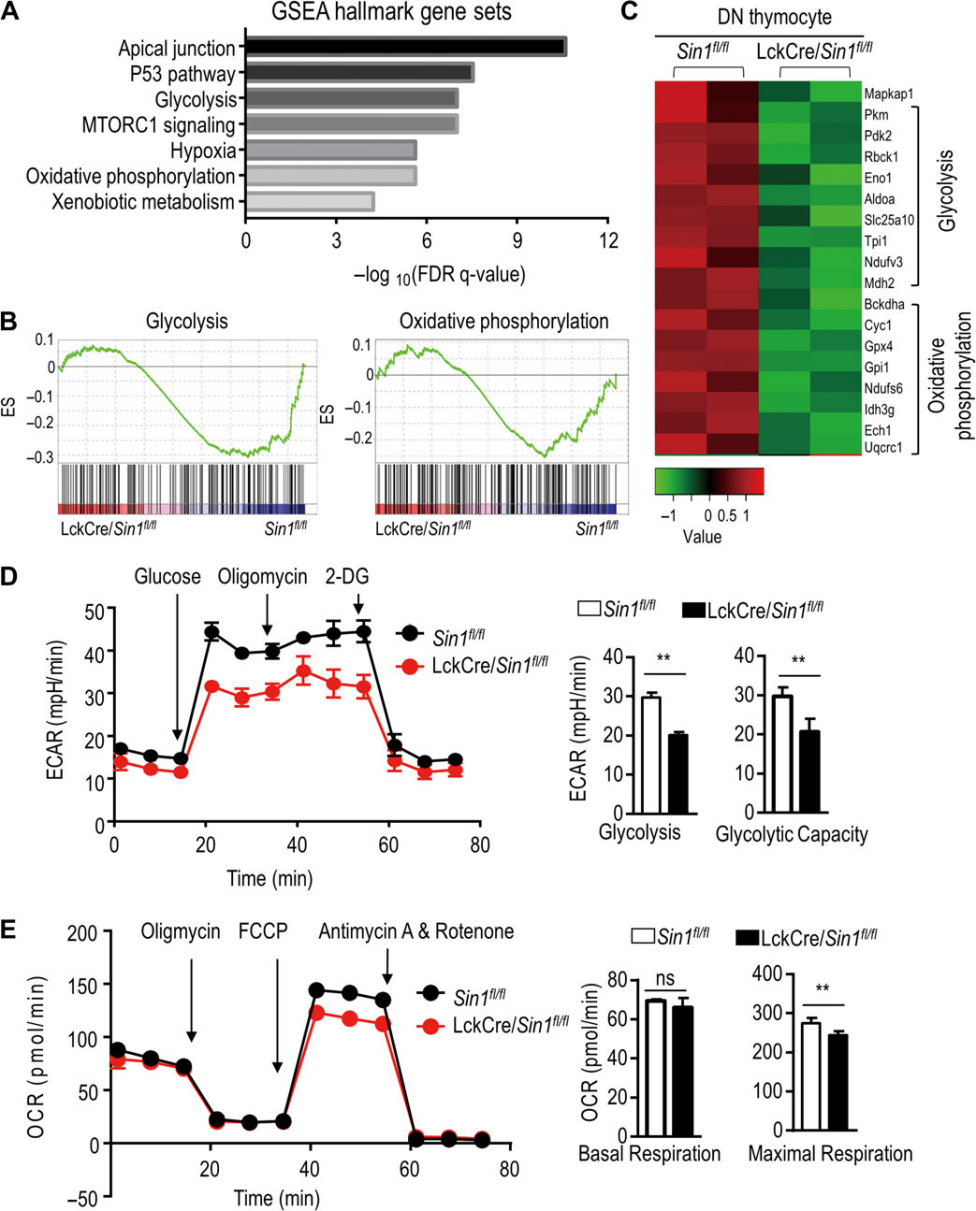
Sin1 is required for glycolysis and oxidative responses in developing thymocytes. (**A**) Downregulated gene sets in DN thymocytes of Sin1-deficient mice by GSEA analysis based on RNA-seq analysis. RNA was extracted from DN thymocytes that were sorted from the total thymocytes of the *Sin1*^*fl/fl*^ and LckCre/*Sin1*^*fl/fl*^ mice and sequenced on an Illumina Nextseq 500 in a 75-bp paired-end configuration. (**B**) GSEA analysis of differentially expressed genes in DN thymocytes. ES, enrichment score. (**C**) Decreased genes of LckCre/*Sin1*^*fl/fl*^ mice in glycolysis and oxidative phosphorylation. The data were analyzed by R package DESeq2. (**D**) ECAR measurements of DN thymocytes isolated from *Sin1*^*fl/fl*^ mice and LckCre/*Sin1*^*fl/fl*^ mice. The process of ECAR measurement is shown on the left. The right bar graph depicts the results from the left curved chart (*n* = 3). (**E**) OCR measurements of DN thymocytes isolated from *Sin1*^*fl/fl*^ mice and LckCre/*Sin1*^*fl/fl*^ mice. The process of OCR measurement is shown in the left and the right bar graph depicts the results from the left curved chart (*n* = 3). Error bars show mean ± SD. Significance was determined by two-tailed Student’s *t*-test (**P < 0.01; ns, no significant difference).

To understand the underlying molecular mechanisms of Sin1-mediated thymocyte development, we performed RNA-seq analysis using DN thymocytes from LckCre/*Sin1*^*fl/fl*^ and control WT mice. The RNA-seq data analyzed by GSEA, which include both down-regulated and up-regulated GSEA hallmark gene sets or pathways, are shown in Figure 4A and Supplementary Figure S6. These data suggest that both glycolysis and oxidative phosphorylation gene sets are down-regulated in *Sin1* KO DN thymocytes. Consistently, the transcription of both oxidative phosphorylation and glycolysis gene sets was significantly decreased after Sin1 deletion (Figure 4B and C). Sin1 is reported to interact with MEKK2 and associate with MAPK signal pathway (Cheng et al., 2005). However, we found that the transcription of the genes targeted by MAPK signal pathway is not significantly changed (Supplementary Figure S7). Given that the Sin1–mTORC2 signals are pivotal for the cell growth and metabolism, these data suggest that the defects in DN thymocyte development in *Sin1* T lineage KO mice may not be MAPK related but rather linked to the mTORC2-mediated metabolic regulation.

To examine this potential metabolic regulation of DN thymocytes by Sin1, the extra-cellular acidification rate (ECAR) and the oxygen consumption rate (OCR) were measured using an XF96 Extracellular Flux Analyzer (Seahorse Bioscience). We found that the glycolytic capacity of *Sin1* KO DN thymocytes was significantly lower than that of the WT DN thymocytes (Figure 4D), and the capacity of maximal respiration of DN thymocytes was also decreased in *Sin1* KO DN thymocytes (Figure 4E). These data suggest that Sin1 is critically involved in regulating the metabolic demands for thymocyte growth and development.

### Sin1 regulates PKM2 expression in the DN thymocytes

**Figure 5:**
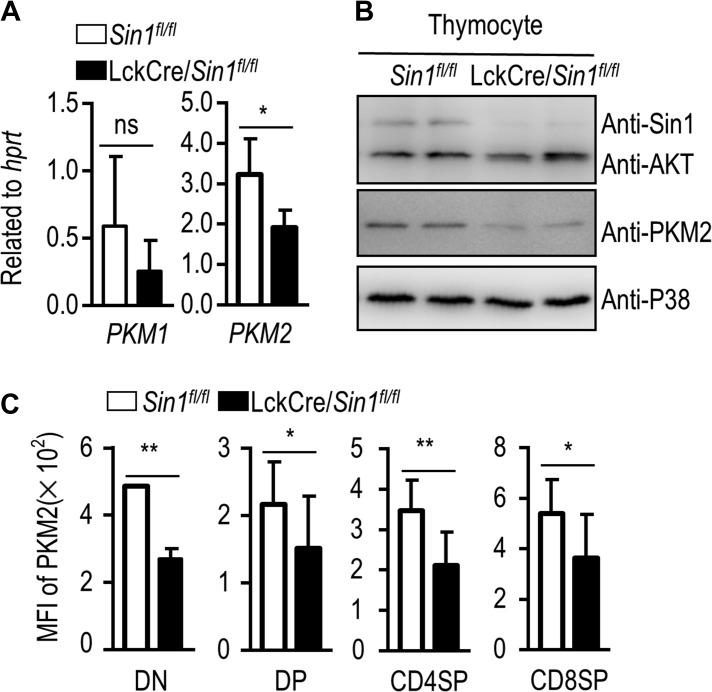
Sin1 regulates PKM2 expression level in developing thymocytes. (**A**) *Pkm* gene transcription in DN thymocytes. RNA was extracted from DN stage thymocytes, and *hprt* serves as control. The data were collected from three pairs of 6- to 8-week-old *Sin1*^*fl/fl*^ mice and LckCre/*Sin1*^*fl/fl*^ mice. (**B**) Immune blot assay for PKM2 expression in thymocytes. Thymocytes were freshly isolated from *Sin1*^*fl/fl*^ mice and LckCre/*Sin1*^*fl/fl*^ mice. Whole-cell extracts were prepared for immunoblotting of PKM2 expression, and P38 expression is served as a loading control. (**C**) PKM2 expression in thymocyte subsets. Mean fluorescence intensity (MFI) of PKM2 in thymocytes was detected by FACS. The data shown are from three pairs of 6- to 8-week-old *Sin1*^*fl/fl*^ mice and LckCre/*Sin1*^*fl/fl*^ mice. Error bars show mean ± SD. Significance was determined by two-tailed Student’s *t*-test (*P < 0.05; **P < 0.01; ns, no significant difference).

The reduced glycolysis and oxidative capacity prompted us to search for differentially expressed genes associated with the metabolism alteration from our RNA-seq data from the WT and *Sin1* KO DN thymocytes. Among them, we found that PKM2 expression was dramatically and consistently reduced in two independent pairs of samples tested (Figure 4C). Indeed, this differential PKM2 expression was confirmed by Q-PCR (Figure 5A). To further verify that the reduced expression is functional relevant, we prepared cell lysates from freshly isolated Sin1-deficient and WT thymocytes for immune blot and verified that the protein level of PKM2 was also significantly reduced in Sin1-deficient thymocytes as compared to that in WT thymocytes (Figure 5B and C). Importantly, the protein level of PKM2 decreased most dramatically in Sin1-deficient DN thymocytes (Figure 5C). Together, these data suggest that the Sin1-mediated metabolic regulation of DN thymocyte development may be mediated by a high PKM2 activity.

### Sin1 controls PKM2 expression through phosphorylation and activation of AKT

**Figure 6:**
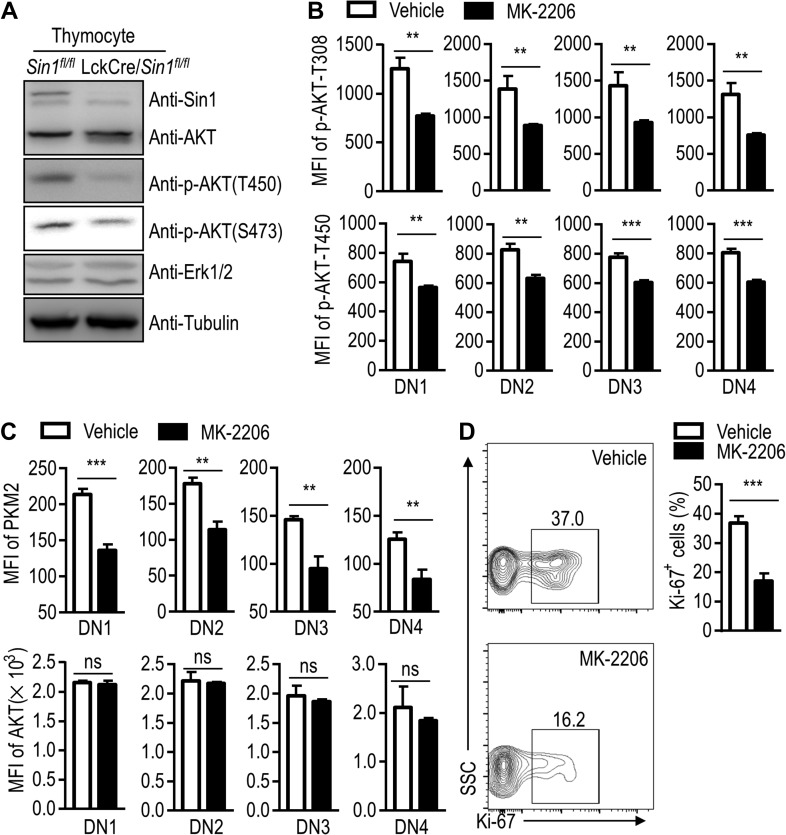
Sin1-dependent PKM2 expression requires AKT activation. (**A**) Immune blot assay of AKT phosphorylation in thymocytes from 6- to 8-week-old *Sin1*^*fl/fl*^ mice and LckCre/*Sin1*^*fl/fl*^ mice. Erk1/2 and Tubulin served as controls. (**B**) Bar graphs of the MFI of AKT phosphorylation in thymocytes. Thymocytes were isolated from 7-week-old WT mice treated with vehicle or MK-2206 (*n* = 3). (**C**) Bar graphs of the MFI of PKM2 in thymocytes (*n* = 3). Thymocytes were isolated as in **B**. The MFI of AKT served as the control. (**D**) Ki-67 expression in DN thymocytes. Thymocytes were isolated and stained with surface makers as in **B**, then followed by anti-Ki-67 antibody staining. FACS graphs are shown on the left, and the numbers in the outlined areas indicate the percentage of positive cells in each area. The right bar graph depicts the results from the FACS data shown on the left (*n* = 3). Error bars show mean ± SD. Significance was determined by two-tailed Student’s *t-*test (**P < 0.01; ***P < 0.001; ns, no significant difference).

Sin1–mTORC2 is an important upstream activator of AKT, an essential mediator of a large spectrum of extracellular signals including growth factors and developmental cues in regulating glucose metabolism and cell proliferation (Jacinto et al., 2006; Hagiwara et al., 2012; Hao et al., 2014; Kocalis et al., 2014; Liu et al., 2014). Interestingly, it has been well determined that growth factors, such as IGF1 and insulin, can upregulate PKM2 expression through AKT activation (Asai et al., 2003; Salani et al., 2015). Given Sin1–mTORC2 is a master activator of AKT, it is not surprise to find that the AKT Ser473 phosphorylation was abolished in Sin1-deficient thymocytes (Figure 6A). This result suggests that AKT may regulate PKM2 expression in thymocytes. To test this possibility, we treated WT mice (5-week old) with an AKT inhibitor MK-2206 (300 μg/kg/day by i.p. injection for 10 days). Compared with the vehicle-treated mice, the phosphorylation of AKT was clearly inhibited. Consistent with our prediction, the expression of PKM2 was also significantly reduced in the DN thymocytes from MK-2206-treated mice (Figure 6B and C). Furthermore, cell proliferation of DN thymocytes as measured by Ki-67-labeling was also significantly decreased after the MK-2206 treatment (Figure 6D). We also used AKT1/2 WT and double KO (AKT1/2^−/−^) MEF cells to verify the function of AKT in the regulation of PKM2 expression. A lower level of PKM2 in AKT1/2^−/−^ MEF cells than that in WT cells was observed (Supplementary Figure S8A). AKT inhibitor treatment impaired PKM2 protein expression in WT MEF cells, but not AKT1/2^−/−^ MEF cells (Supplementary Figure S8B and C). Together, these results suggest that Sin1–mTORC2 regulates PKM2 expression through AKT activation.

### Sin1 mediates PKM2 upregulation by promoting PPAR-γ nuclear translocation

**Figure 7:**
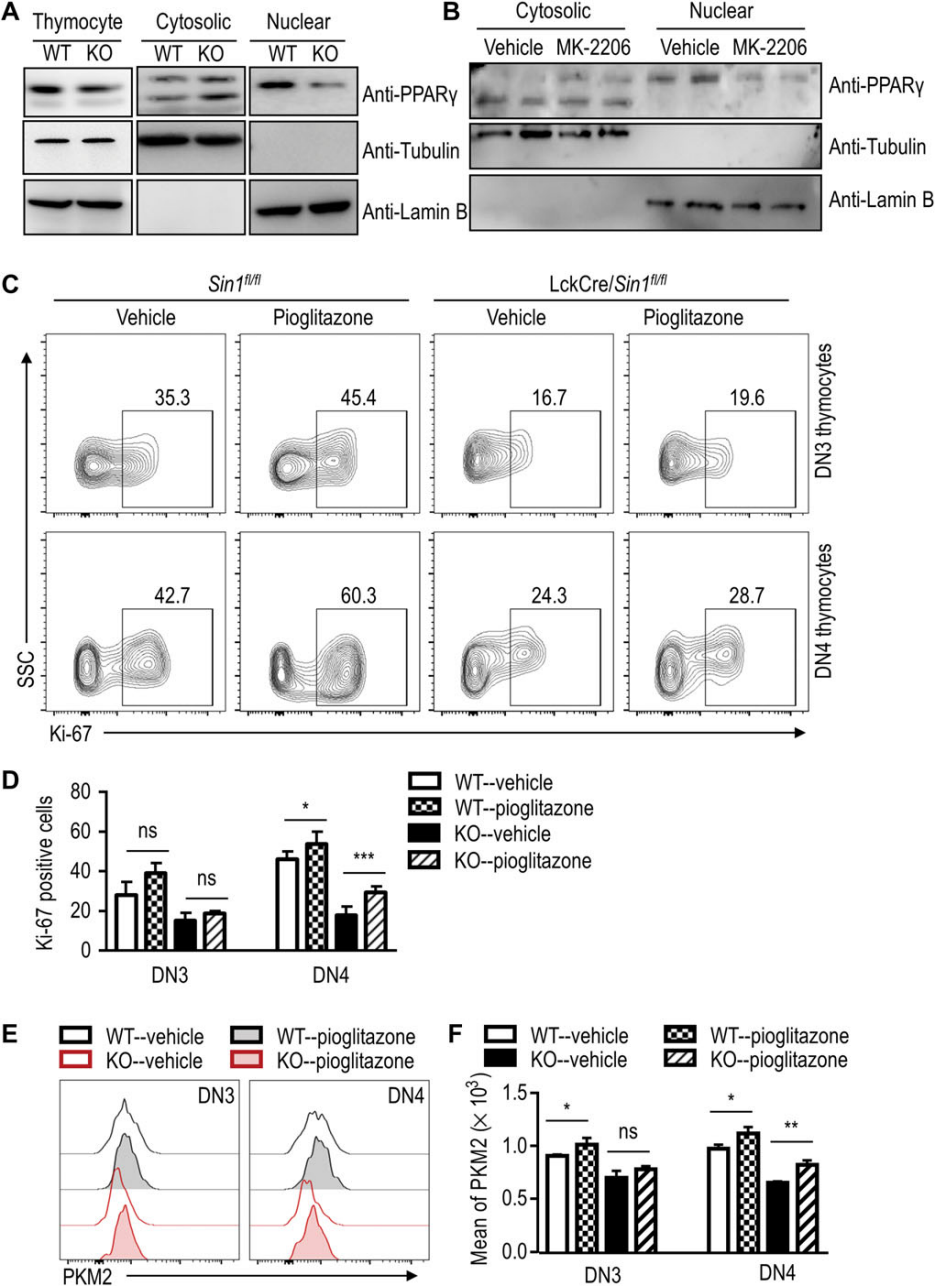
The Sin1-dependent PKM2 expression in developing thymocytes requires an AKT-controlled PPAR-γ nuclear translocation. (**A**) The expression of PPAR-γ in thymocytes from *Sin1*^*fl/fl*^ mice and LckCre/*Sin1*^*fl/fl*^ mice. PPAR-γ was detected by immune blot, with Tubulin and Lamin B served as control. (**B**) The expression of PPAR-γ in nucleus and cytoplasm of thymocytes treated with MK-2206 or vehicle *in vivo*. Tubulin and Lamin B served as control. (**C**) Ki-67 expression in DN thymocytes from mice treated with vehicle or pioglitazone. Thymocytes were isolated from *Sin1*^*fl/fl*^ mice and LckCre/*Sin1*^*fl/fl*^ mice treated with vehicle or pioglitazone. The Ki-67-positive cells were shown in thymocyte subsets, which were distinguished as described before. The numbers in the outlined areas indicate the percentage of cells in each area. (**D**) The statistical analysis of the percentage of Ki-67-positive cells in DN3 and DN4 thymocytes from the results shown in **C** (*n* = 3). (**E**) PKM2 expression in DN thymocytes. Thymocytes were isolated as described in **C**, and the MFI of PKM2 was shown in DN3 and DN4 thymocytes from mice treated with vehicle or pioglitazone. (**F**) The FACS quantification of PKM2 MFI. The data were based on the FACS results in **E**. Error bars show mean ± SD, *n* = 3. Significance was determined by two-tailed Student’s *t*-test (*P < 0.05; **P < 0.01; ***P < 0.001; ns, no significant difference).

It was reported previously that AKT could regulate PKM2 expression by promoting the activation of PPAR-γ, a known transcription activator of PKM2 (Panasyuk et al., 2012). These studies suggest that the reduced PKM2 transcription in Sin1-deficient DN thymocytes could be due to reduced PPAR-γ in the nucleus. To investigate this possibility, we determined the cytosolic and nuclear protein levels of PPAR-γ in WT and Sin1-deficient thymocytes. As shown in Figure 7A, a slight increase of cytosolic PPAR-γ protein level and a corresponding decrease of nuclear PPAR-γ protein level in Sin1-deficient thymocytes was observed, although WT and Sin1-deficient thymocytes had a similar level of total PPAR-γ. These data indicated that Sin1-deficient thymocytes had lower levels of nuclear PPAR-γ than that in WT thymocytes. Consistently, we show that the PPAR-γ nuclear translocation could be inhibited in the DN thymocytes in WT mice by given an AKT inhibitor MK-2206 (Figure 7B).

To further link the impaired DN thymocyte development to the reduced activity of PPAR-γ in Sin1-deficient mice, we treated the LckCre/*Sin1*^*fl/fl*^ mice and the WT littermate control with a known PPAR-γ activator pioglitazone. After 2-week treatment, proliferation of DN thymocytes was analyzed. As shown in Figure 7C, cell proliferation, as measured for the ratio of Ki-67-positive cells, of the Sin1-deficient DN3 and DN4 thymocytes was significantly augmented in pioglitazone-treated mice as compared to that of vehicle-treated mice (Figure 7C and D). Importantly, the expression of PKM2 was also restored in pioglitazone-treated thymocytes (Figure 7E and F). These results demonstrate that Sin1 upregulates PKM2 expression by promoting an AKT-dependent PPAR-γ nuclear translocation.

### Administration of a PKM2 agonist partially rescues Sin1-deficient DN thymocyte development

**Figure 8:**
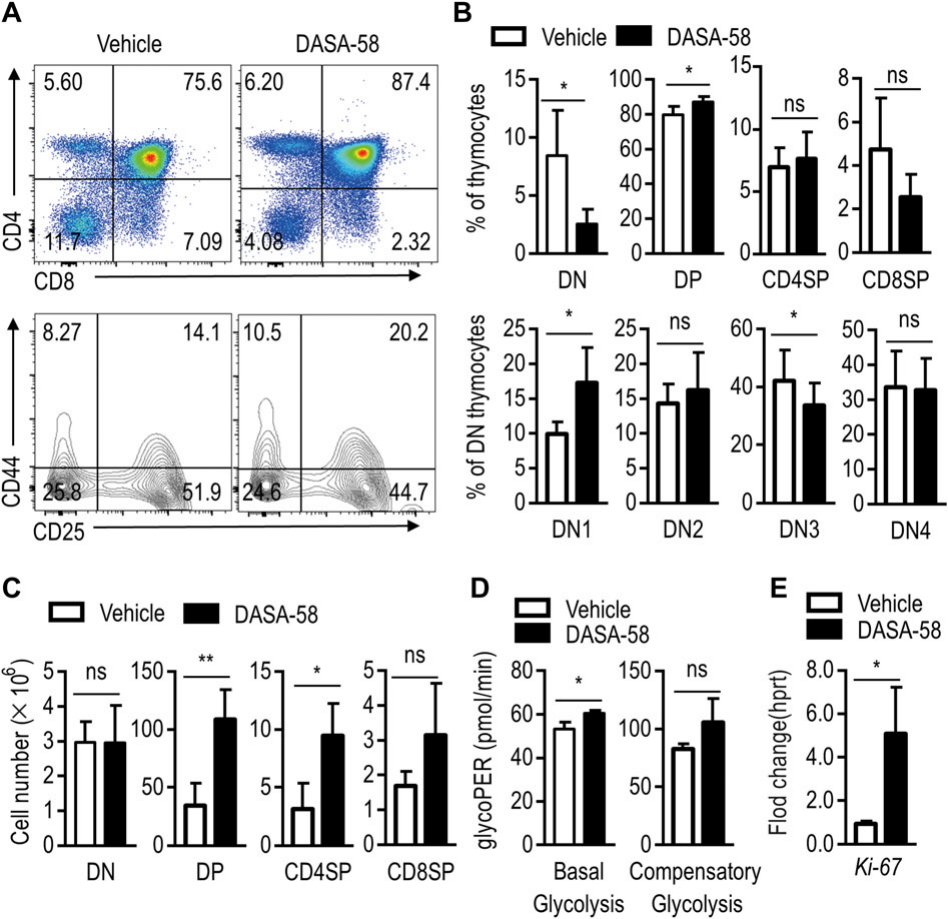
Sin1-deficient thymocyte development could be partially rescued by *in vivo* treatment with a PKM2 agonist. (**A**) Surface staining of total thymocytes from LckCre/*Sin1*^*fl/fl*^ mice treated with vehicle or the PKM2 activator DASA-58. Upper panels are CD4 and CD8 staining. Lower panels are gated on the CD4 and CD8 DN subset for further CD44 and CD25 expression analyses. Numbers in the panels show the relative percentage of cells in that area. (**B**) Quantification of DN, DP, CD4, or CD8 SP thymocyte subsets determined by FACS in **A** (*n* = 3). (**C**) Summary graphs of thymocyte subset cell number. The data were summarized from **A** and **B**. (**D**) ECAR measurements of DN thymocytes isolated from mice described in **A**. (**E**) Ki-67 RNA levels in mice thymocytes. Total RNA was extracted from thymocytes of LckCre/*Sin1*^*fl/fl*^ mice, which were treated with DASA-58 or vehicle for 3 h *in vitro* (*n* = 3). Error bars show mean ± SD. Significance was determined by two-tailed Student’s *t*-test (*P < 0.05; **P < 0.01; ns, no significant difference).

We next examine the role of PKM2 in Sin1 dependent DN thymocyte development, we treated LckCre/*Sin1*^*fl/fl*^ mice with a well-characterized PKM2 activator DASA-58 for 10 days. As shown in Figure 8A and B, DASA-58 treatment led to a partial rescue of DN3 to DN4 development in LckCre/*Sin1*^*fl/fl*^ mice. Moreover, we found that the cell numbers of DP, CD4SP, and CD8SP were all increased after DASA-58 treatment in LckCre/*Sin1*^*fl/fl*^ mice (Figure 8C). Consistently, the DASA-58 treatment also led to increased level of basal glycolysis and proliferation of DN thymocytes (Figure 8D and E). Together, these results confirm that PKM2 is a key downstream mediator of the Sin1 signal in early thymocyte development.

## Discussion

In this study, we reveal a novel role of Sin1 in the regulation of cellular metabolism in early thymocyte development. We show that Sin1, via mTORC2, promotes DN thymocyte proliferation through augmenting the transcriptional level of PKM2, a key enzyme for the final rate-limiting step of glycolysis by catalyzing the transfer of phosphoenolpyruvate (PEP) to pyruvate. Consistently, *in vitro* and *in vivo* treatments with PKM2 activator confirm that PKM2 is responsible for mediating the Sin1–mTORC2 signaling in thymocyte development. At the molecular level, we show that the Sin1-mediated PKM2 expression regulation is mediated by a Sin1–mTORC2–AKT-dependent PPAR-γ nuclear translocation. This Sin1–AKT–PPAR-γ-controlled PKM2 expression contributes to DN thymocyte proliferation and metabolism (Supplementary Figure S9). Together, our work reveals a previously unknown function of Sin1–mTORC2 signaling in the metabolic regulation of early thymocyte development.

A previous study using *Rictor* cKO mice suggested a critical role of mTORC2 in early thymocyte development (Tang et al., 2012). Using Sin1-deficient FL-HSC reconstituted mice, we showed previously a modest augmentation of Treg in the thymus, and an increased percentage of DN thymocytes but with no other major abnormality in T cell lineage (Chang et al., 2012). Using an *in vitro* OP9-fetal liver-HSC co-culture system, we did not observe defects in the thymocyte development in this system (Chang et al., 2012). However, using the conditional Sin1-deletion mice in T cell lineage, we show a very clear DN developmental blockade at this stage, but not at the later stage. Together these data suggest that Sin1–mTORC2 is a key regulator of early thymocyte development (Lee et al., 2012; Tang et al., 2012; Chou et al., 2014).

Although both Sin1 and Rictor are key essential components of mTORC2, Sin1 may also regulate non-mTORC2 functions (Wilkinson et al., 1999; Cheng et al., 2005; Schroder et al., 2007), especially the MAPK signaling. Although MAPKs are also important for regulating the thymocyte development, the major MAPK signature genes were not enriched in the down-regulated gene sets or up-regulated gene sets (Figure 4A and Supplementary Figure S6) suggesting that at the DN stage, the Sin1-mediated function may not involve the MAPK signaling. Furthermore, the level of MAPK downstream target genes in the WT and Sin1-deficient DN thymocytes were comparable (Supplementary Figure S7). Together these data suggest that the major Sin1 function is likely mediated through the mTORC2 pathway.

Interesting, although defect thymocyte proliferation and development was observed in both Sin1- and Rictor-deficient mice, there are differences in the phenotypes of these *Sin1-* and *Rictor*-target T cells. We found that the expression of cell surface markers TCRβ, Notch1, Notch3, and CD127 on the developing thymocytes was not affected in *Sin1* KO thymocytes but was reported to be attenuated in Rictor-deficient thymocytes (Chou et al., 2014). In another study, it was reported that Cd4Cre-mediated *Rictor* deletion resulted in reduced thymocyte cell number (Wei et al., 2014), while in our system, the Cd4Cre-mediated *Sin1* deletion has no impact on the total number of thymocytes, or thymocyte development at DN, DP, or SP stages. Currently, we do not know the reasons for these differences but could be due to the different mouse strains used in the studies. Of note, although mTORC2 is highly related to cellular metabolism, the link between the mTORC2 signaling and thymocyte metabolism during early T cell development is still unclear.

Energy supplement plays a pivotal role in regulating thymocyte proliferation. Previous studies have demonstrated that thymic cellularity is increased through DN thymocyte proliferation, and 85% of the energy consumed for thymocyte proliferation originates from glycolysis (Brand and Hermfisse, 1997). Additionally, it has been reported that glycolysis is augmented during thymocyte proliferation at the DN3 to DN4 stage (Sandy et al., 2012; Buck et al., 2015). How glycolysis in thymocytes is regulated at this stage remains unclear. Our current study reveals that the expression of many glycolysis regulating genes is markedly downregulated in Sin1-deficient DN thymocytes (Figure 4A and C). Consistently, glycolysis capacity in DN thymocytes was markedly impaired in Sin1-deficient mice. Our findings suggest that Sin1–mTORC2 participates in the glycolytic regulation of DN thymocytes, which likely explain the defect in DN3–DN4 transition. In addition to the impaired glycolysis, we found that oxidative phosphorylation (OXPHOS)-related genes and OCR were also downregulated in the Sin1-deficient DN thymocytes. We still do not know the mechanism of these Sin1 regulated oxidative phosphorylation in early T cell development. Given the importance of OXPHOS-related genes in cell growth and metabolism, future investigation on whether or how the Sin1–mTORC2-regulated OXPHOS contributes to the early T cell development may be fruitful.

Among the metabolically regulated genes that were found impaired in Sin1-deficient DN thymocytes, PKM2 was further confirmed and verified as a target of Sin1. PKM2 is predominantly localized in the cytosol as a key enzyme for glycolysis; however, it has also been shown to function as a protein kinase and transcriptional co-activator in the nucleus (Israelsen et al., 2013; Jiang et al., 2014; Lunt et al., 2015). Little is known about the role and regulation of PKM2 in T cell development. In this study, we identify PKM2 as a key target of Sin1–mTORC2 signaling and reveal an essential function of PKM2 in the early stages of thymocyte development. We provide several pieces of evidence to support this conclusion. First, by utilizing an AKT inhibitor to block the Sin1–mTORC2–AKT signaling in DN thymocytes, we confirm that PKM2 expression was down-regulated. Secondly, we utilized a highly specific small-molecule activator DASA-58 to augment the activity of PKM2 in Sin1-deficient mice, and showed that this treatment restored Sin1-deficient thymocyte metabolism and rescued early thymocyte development. Finally, we used pioglitazone, a potent agonist of PPAR-γ, which is known to promote hepatocyte glycolysis and proliferation via controlling PKM2 expression (Panasyuk et al., 2012), to confirm that activation of PPAR-γ could partially restore PKM2 expression and rescue the proliferation and development of Sin1-deficient DN thymocytes.

How does Sin1–mTORC2 control PKM2 expression at the molecular level has not been well studied before. We show that in Sin1-deficient DN thymocytes, the Sin1–mTORC2-mediated AKT activation was impaired. AKT has been shown to control the transcriptional levels and nuclear translocation of PPAR-γ (Panasyuk et al., 2012). In this study, we found that Sin1 could mediate PPAR-γ nuclear translocation to promote PKM2 expression in the DN thymocytes. However, the mRNA level of PPAR-γ in the Sin1-deficient DN thymocytes was not affected (data not shown) suggesting that AKT may control PPAR-γ slightly different in different tissues.

Our RNAseq data revealed that Sin1 deficiency results in the down-regulation of a series of genes in DN thymocytes, including PKM2, pyruvate dehydrogenase kinase isoform 2 (PDK2), triose phosphate isomerase (Tpi1), and Enolase 1 (Eno1). These proteins have been proved to be involved in the regulation of glycolysis-mediated cell proliferation. In our current report, we identify PKM2 as a novel and crucial Sin1 effector in promoting DN thymocyte development and metabolism. Whether Sin1–mTORC2 signaling regulates DN thymocyte development and metabolism via controlling PDK2, Tpi1, or/and Eno1 expression remains unknown and these studies may prove to be fruitful in the future.

In conclusion, this study has identified Sin1 as an important regulator of DN stage thymocyte metabolism, proliferation, and development. We unravel a previously unknown signaling cascade involving the Sin1–mTORC2–AKT–PPAR-γ signaling to upregulate PKM2, a key metabolic regulator essential for cell proliferation. These findings reveal an additional function of Sin1–mTORC2 in the immune-metabolic regulation of thymocyte DN3–DN4 transition, and may provide new strategies and potential targets for the clinical treatment of T cell lymphopenia or T cell lymphoma.

## Materials and methods

### Mice

To generate a conditional *Sin1 *knockout mouse model, we purchased an ES cell line containing the *Sin1* target allele from EUCOMM (European Conditional Mouse Mutagenesis Program), which contains loxP and Frt sites and selection genes (*lacZ* and *Neomycin*) (Supplementary Figure S1A). The conditional knockout mice were generated by breeding to various tissue-specific Cre-expressing mouse lines. Briefly, the ES cells were first expanded and verified by PCR (Supplementary Figure S1B), and then injected into blastocysts for chimeric offspring. F0 generation chimeric mice were screened by PCR for mice containing the *Sin1* target allele (Supplementary Figure S1C). Positive chimeras were bred with WT mice to obtain mice with germline transmission of the *Sin1* target allele. These mice with targeted allele were next bred to Flp expression mice [B6.SJL-Tg (ACTFLPe) 9205Dym/J] to delete the *lacZ* and *Neomycin* cassette via a *flp*-mediated recombination (Supplementary Figure S1A), resulting in the generation of mice with a floxed exon 4 at the *Sin1* allele, which we designated as *Sin1*^*fl/+*^ mice. These *Sin1*^*fl*+^ mice were backcrossed more than nine generations onto the C57BL/6 background, and self-crossed to generate *Sin1*^*fl/fl*^ mice, which grow and develop normally as the WT littermate mice. After the *Sin1*^*fl/fl*^ mice were crossed with a tamoxifen (TM) inducible *ROSA26*-Cre-ER^T2^ mouse line to generate *ROSA26*-Cre-ER^T2^/*Sin1*^*fl/fl*^ (referred to as ERCre*/Sin1*^*fl/fl*^*mice*), control (*Sin1*^*fl/fl*^) or ERCre/*Sin1*^*fl/fl*^ mice (6–8 weeks old) were injected intraperitoneally tamoxifen (TM) daily (2 mg/dose, 5 doses) to inducibly delete *Sin1* (Supplementary Figure S1A). Sin1 deletion was confirmed by PCR and immune blot in various tissue and organs including thymus and spleen (Supplementary Figure S1D). Although embryonic deletion of Sin1 in ERCre/*Sin1*^*fl/fl*^ mice by TM injection is lethal (data not shown), no obvious growth defects or global appearance abnormality were found in TM-induced ERCre/*Sin1*^*fl/fl*^ mice at the adult stage as compared with the control *Sin1*^*fl/fl*^ mice, which we used as WT control mice throughout the study. *Sin1*^*fl/fl*^ mice were also crossed with LckCre or Cd4Cre mice in the C57BL/6 background (The Jackson Laboratory) to generate T lineage *Sin1 *knockout mice (LckCre/*Sin1*^*fl/fl*^ or Cd4Cre/*Sin1*^*fl/fl*^). PCR genotyping as well as immune blot assay were performed to verify that Sin1 could be deleted effectively (Supplementary Figure S1A and B). Mice were maintained in a specific pathogen free (SPF) facility and all mouse experiments were conducted in accordance with protocols approved by the Institutional Animal Care and Use Committee of Shanghai Jiao Tong University School of Medicine.

### Antibodies and reagents

Sin1-specific antibody was prepared by immunizing rabbits, and the antibody was further affinity purified (Cheng et al., 2005). The antibodies used in the immune blots for AKT (Cat. number 9272), Erk1/2 (Cat. number 9102), P38 (Cat. number 9212), p-AKT(T308) (Cat. number 2965), p-AKT(T450) (Cat. number 9267), p-AKT(S473) (Cat. number 4060), and PKM2 (Cat. number 4053) were purchased from Cell Signaling Technology, and the antibody used in the immune blot for PPAR-γ (Cat. number sc-166731) was purchased from Santa Cruz Biotechnology, Inc. The antibody used in the flow cytometry (FACS) for PKM2 (Cat. number ab150377) was obtained from Abcam. The inhibitor MK-2206 (Cat. number s1078), PKM2 activator DASA-58 (Cat. number s7928), and pioglitazone (Cat. number s2590) were purchased from Selleck.

### BM chimaeras

For the transfer model, CD45.1^+/+^ mice were treated with 8 grays of irradiation and received 2 × 10^6^ BM cells from *Sin1*^*fl/fl*^ mice or ERCre/*Sin1*^*fl/fl*^ mice via tail vein injection. In the competitive model, irradiated CD45.1^+^CD45.2^+^ mice received a mixture of BM cells from C57BL/6 CD45.1 mice (CD45.1^+^) and *Sin1*^*fl/fl*^ mice or ERCre/*Sin1*^*fl/fl*^ mice (CD45.2^+^) at a 1:1 ratio (2 × 10^6^ cells in total). All mice were of the C57BL/6 background, and BM chimaeras were maintained in a specific pathogen-free environment.

### Flow cytometry (FACS)

Suspensions of thymocytes were stained for surface antigens in cold FACS buffer (1× PBS pH 7.4 + 2% FBS) containing the indicated specifically conjugated antibodies and subjected to FACS and cell sorting using an BD LSRFortessa^TM^-X20 (BD) and a FACS AriaIII (BD) flow cytometer, respectively. The data were analyzed with FlowJo software. Intracellular cytokine staining of p-AKT, AKT, and PKM2 was carried out as previously described (Chang et al., 2012). BrdU (BD) and Ki-67 (eBioscience) staining was carried out according to the manufacturer’s instructions.

### Immune blot

Freshly isolated thymocytes were washed with ice-cold PBS and then lysed in cold RIPA buffer with fresh phosphatase inhibitor cocktail. The total cell lysates were resolved by SDS-PAGE and blotted with the associated antibodies. The signals were visualized using a luminescent image analyzer (GE) and ImageQuant LAS 4000 software.

### Q-PCR

Thymocytes were lysed in TRIzol (Invitrogen) and total RNA was purified by isopropanol precipitation. The total RNA was reverse transcribed according to the manufacturer’s instructions (TaKaRa). Quantitative RT-PCR was performed with an iQ5 multicolor RT-PCR detection system (ABI) using the SYBR Green Supermix PCR master mix kit (Life).

### Thymocyte metabolic assay

DN thymocyte single cell suspensions were isolated from *Sin1*^*fl/fl*^ and LckCre/*Sin1*^*fl/fl*^ mice. Thymocytes were plated on Seahorse cell culture plates pre-coated with Cell-Tak (BD Biosciences) (5 × 10^5^/well). The OCR and ECAR for thymocyte metabolism were analyzed on an XF96 Extracellular Flux Analyzer (Seahorse Bioscience) according to the manufacturer’s instructions.

### RNA-seq

DN thymocytes were purified by sorting from *Sin1*^*fl/fl*^ and LckCre/*Sin1*^*fl/fl*^ mice, and DN thymocyte RNA was then isolated. cDNA libraries were prepared using the Illumina Truseq stranded mRNA kit according to the manufacturer’s instructions. The libraries were then sequenced on an Illumina Nextseq 500 in a 75-bp paired-end configuration. Approximately, 20 million read pairs per sample were produced. The data were then processed using TOPHAT software (Kim et al., 2013) and HTseq (Anders et al., 2015) and then analyzed with R package DESeq2 (Love et al., 2014). Gene enrichment analysis was carried out using GSEA (Subramanian et al., 2005).

### Statistical analysis

Statistical analysis was performed using Prism software (GraphPad Software). Two-tailed unpaired Student’s *t*-tests were performed, *P*-values < 0.05 were considered significant, and the level of significance was indicated as follows: *P < 0.05; **P < 0.01, ***P < 0.001, ****P < 0.0001.

## Supplementary Material

Supplementary Data
