# Supplementary material for: Metabolic regulation of T cell development by Sin1–mTORC2 is mediated by pyruvate kinase M2
Source: J Mol Cell Biol. 2018 Nov 14;11(2):93–106. doi: 10.1093/jmcb/mjy065 (PMC6392101; doi:10.1093/jmcb/mjy065)
Supplement: Supplementary Data [file mjy065_supplementary_material.pdf]

Supplementary Material

Metabolic regulation of T cell development by Sin1-mTORC2 is mediated by pyruvate kinase M2

Xinxing Ouyang[1], Yuheng Han[1], Guojun Qu[1], Man Li[1], Omotooke Arojo[2], Hongxiang Sun[1], Xiaobo Liu[1], Dou Liu[2], Lei Chen[1], Qiang Zou[1,*], and Bing Su[1,2,*]

[1]Shanghai Institute of Immunology, Department of Immunology and Microbiology, Key Laboratory of Cell Differentiation and Apoptosis of Chinese Ministry of Education, Shanghai Jiao Tong University School of Medicine, Shanghai 200025, China
[2]Department of Immunobiology and the Vascular Biology and Therapeutics Program, Yale University School of Medicine, 333 Cedar Street, New Haven, Connecticut 06520, USA

[*]Correspondence should be addressed to: Qiang Zou (qzou1984@sjtu.edu.cn) or Bing Su (bingsu@sjtu.edu.cn).

Contents

I. Supplementary Figures.. 3

Supplementary Figure S1. Generation of conditional knockout mice with targeted mutation of *Sin1* 3

Supplementary Figure S2. Mice reconstituted by Sin1 KO BM show defects in thymocyte development.. 5

Supplementary Figure S3. T cell developmental defects caused by Sin1 deletion are cell intrinsic 7

Supplementary Figure S4. Deletion Sin1 in T cells from LckCre/*Sin1*$^{fl/fl}$ and Cd4Cre/*Sin1*$^{fl/fl}$ mice. 8

Supplementary Figure S5. Sin1 deficiency does not affect CD127 and TCR expression in thymocytes 9

Supplementary Figure S6. Gene sets upregulated in DN thymocytes of Sin1 deficient mice by GSEA analysis 10

Supplementary Figure S7. Sin1 deficiency does not alter the downstream of MAPK signal pathways 11

Supplementary Figure S8. AKT controls PKM2 expression in MEF cells............. 12

Supplementary Figure S9. proposed model illustrating the Sin1-mediated regulation of early thymocyte proliferation and development... 13

II. Supplementary tables.. 14

Supplementary Table S1. Primers used for genotyping. 14

Supplementary Table S2. Primers used for Real time-PCR................................... 14

Supplementary Table S3. Transcriptions significantly more abundant in Sin1 deficient DN thymocytes.. 14

Supplementary Table S4. Transcriptions significantly less abundant in Sin1 deficient DN thymocytes.. 15

I. Supplementary Figures

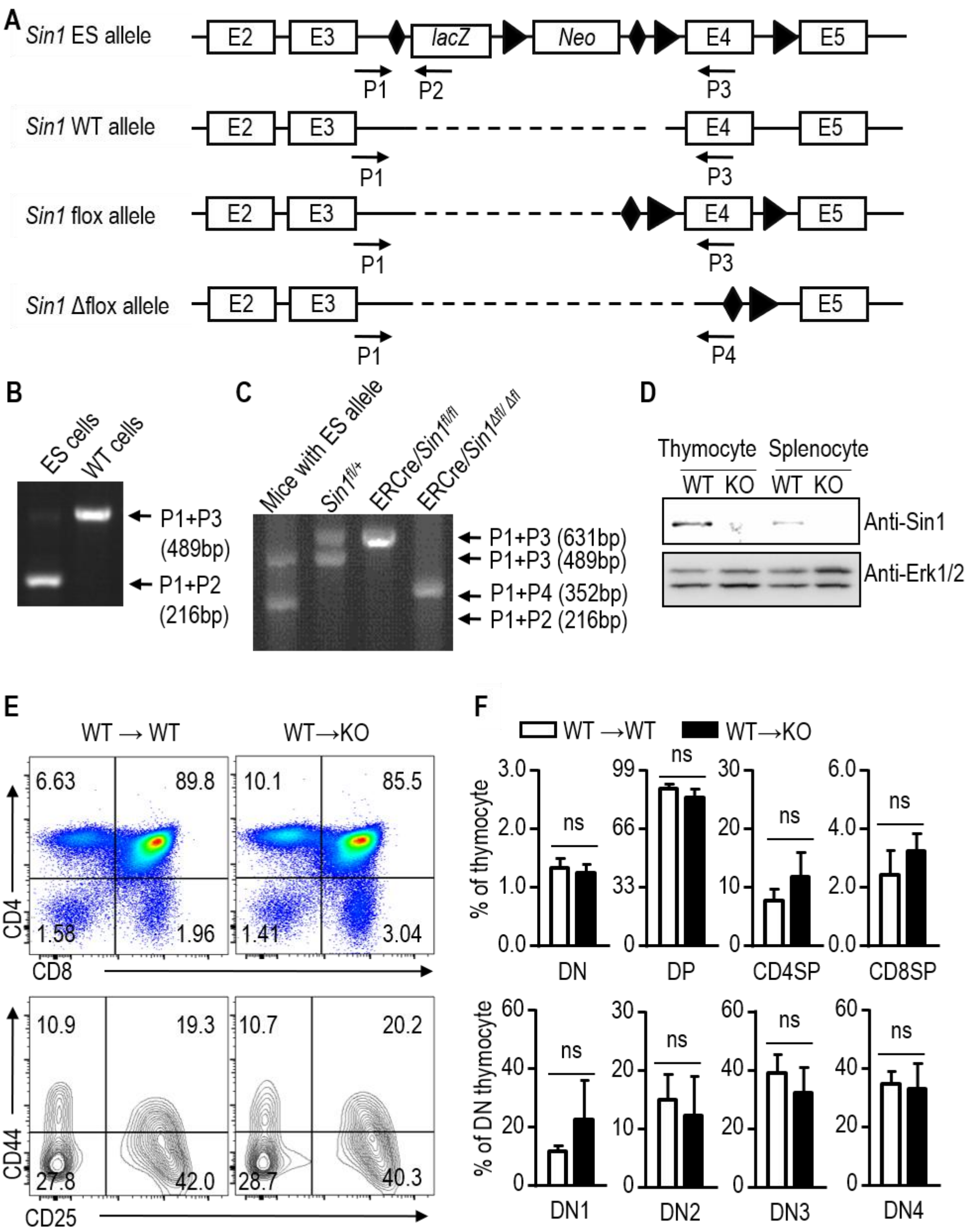

Supplementary Figure S1. Generation of conditional knockout mice with targeted mutation of *Sin1*

(A) A diagram shown the targeted Sin1 allele in ES cells (Sin1 ES allele), Sin1 WT allele, Sin1 flox allele, and Sin1 Δflox allele as indicated. E2, E3, E4 and E5 indicate the exon 2, exon 3, exon 4 and exon 5 of Sin1 gene, respectively. The black diamonds and black triangles indicate the Frt and loxP sites, respectively.
P1(5'-TGTATTAGCCTGCACAAGGAGT-3'),
P2(5'-CAACGGGTTCTTCTGTTAGTCC-3'),

P3(5'-ATGGACTGCTTGCCTGAACT-3') and
P4(5'-ATTGAACTGATGGCGAGCTC-3') are primers used for PCR reactions to distinguish the four types of Sin1 alleles, and their relative locations are also shown in the diagram. (B) PCR analysis of genomic DNA isolated from Sin1 ES cells and WT cells. (C) PCR analysis of genomic DNA isolated from mouse toes. (D) Immune blot assay for Sin1 expression in thymocytes and splenocytes. Thymocytes and splenocytes were freshly isolated from tamoxifen-treated $Sin1^{fl/fl}$ mice and $ERCre/Sin1^{fl/fl}$ mice. Whole cell extracts were prepared for immunoblotting of Sin1 expression, and the Erk1/2 expression is served as a loading control. (E) Surface staining of total thymocytes from lethally irradiated $Sin1^{fl/fl}$ mice (WT) or $ERCre/Sin1^{fl/fl}$ mice (KO) which were reconstituted with bone-marrow cells from wild type (WT) C57BL/6 CD45.1 mice ($CD45.1^+$) with indicated antibodies. Upper panels are CD4 and CD8 staining. Lower panels are gated on the CD4 and CD8 double negative (DN) subset for further CD44 and CD25 expression analyses. Numbers in in the panels show the relative percentage of cells in that area. (F) Quantification of thymocyte subsets determined by FACS as described above in panel E (n=3). Error bars shows mean ± SD. Significance was determined by two-tailed Student's t test (*, $p < 0.05$; ns, no significant).

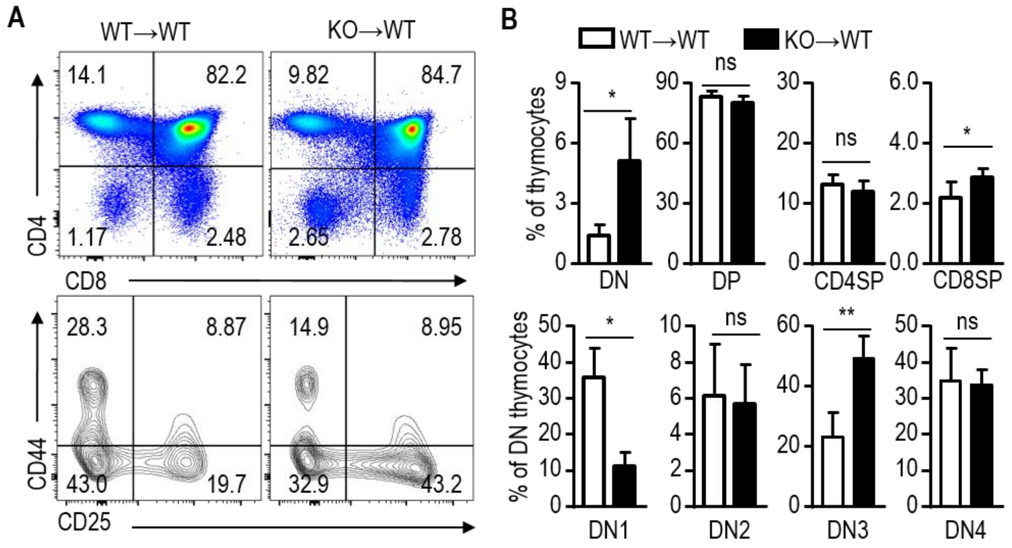

Supplementary Figure S2. Mice reconstituted by Sin1 KO BM show defects in thymocyte development

(A) Surface staining of total thymocytes from irradiated C57BL/6 CD45.1 mice ($CD45.1^+$) that were reconstituted by bone marrow cells isolated from tamoxifen-treated *Sin1*$^{fl/fl}$ mice or *ERCre/Sin1*$^{fl/fl}$ mice($CD45.2^+$). The upper panels show CD4 and CD8 staining, whereas the lower panels show CD44 and CD25 staining of the gated CD4 and CD8 double negative (DN) subsets from the upper panels. Numbers in the panels indicate the percentages of cells in that specific area. (B) Quantification of thymocyte subsets determined by FACS analyses, as described above in (A) (n=4). Error bars shows mean ± SD. Significance was determined by two-tailed Student's *t* test (*, $p < 0.05$; **, $p < 0.01$; ns, no significant difference).

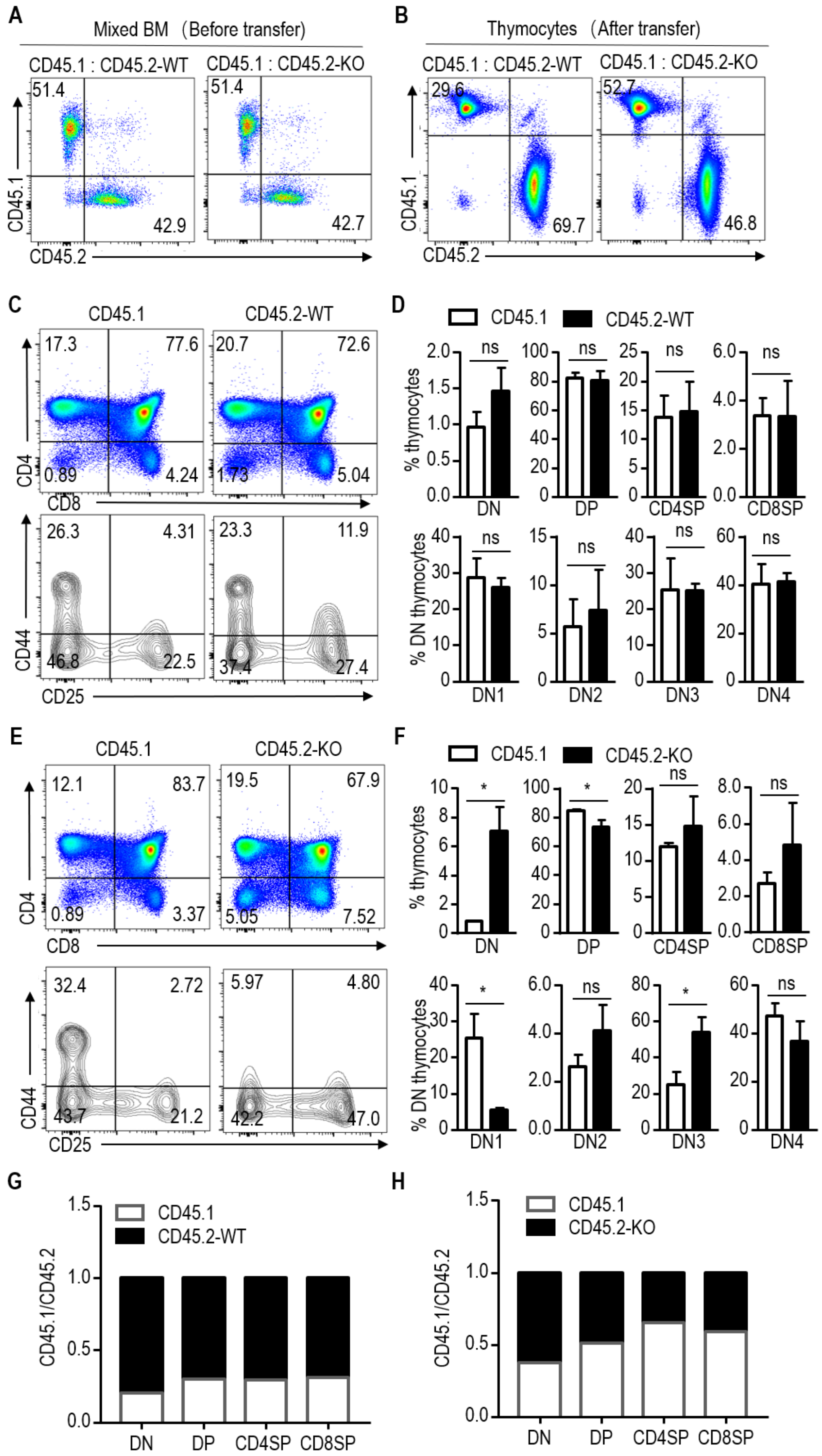
A
Mixed BM (Before transfer)
CD45.1 : CD45.2-WT
CD45.1 : CD45.2-KO
51.4
42.9
51.4
42.7
CD45.1
CD45.2
B
Thymocytes (After transfer)
CD45.1 : CD45.2-WT
CD45.1 : CD45.2-KO
29.6
69.7
52.7
46.8
CD45.1
CD45.2
C
CD45.1
CD45.2-WT
17.3
77.6
0.89
4.24
20.7
72.6
1.73
5.04
CD4
CD8
26.3
4.31
46.8
22.5
23.3
11.9
37.4
27.4
CD44
CD25
D
CD45.1
CD45.2-WT
% thymocytes
% DN thymocytes
ns
DN
DP
CD4SP
CD8SP
DN1
DN2
DN3
DN4
E
CD45.1
CD45.2-KO
12.1
83.7
0.89
3.37
19.5
67.9
5.05
7.52
CD4
CD8
32.4
2.72
43.7
21.2
5.97
4.80
42.2
47.0
CD44
CD25
F
CD45.1
CD45.2-KO
% thymocytes
% DN thymocytes
*
ns
DN
DP
CD4SP
CD8SP
DN1
DN2
DN3
DN4
G
CD45.1
CD45.2-WT
CD45.1/CD45.2
DN
DP
CD4SP
CD8SP
H
CD45.1
CD45.2-KO
CD45.1/CD45.2
DN
DP
CD4SP
CD8SP

Supplementary Figure S3. T cell developmental defects caused by Sin1 deletion are cell intrinsic

(A) FACS results shows Mixed BM before transfer and reconstituted thymocyte population. Bone marrow cells were isolated from C57BL/6 CD45.1 (CD45.1^{+}) and WT(CD45.2^{+}) or Sin1 KO(CD45.2^{+}) mice and mixed at roughly 1:1 ratio. (B) Surface staining of thymocytes from BM reconstituted mice. Thymocytes were shown based on the expression of CD45.1 and CD45.2. (C) Surface staining of thymocytes from the lethally irradiated CD45.1^{+}CD45.2^{+} mice, which were reconstituted by mixed WT (CD45.2^{+}) and C57BL/6 CD45.1 mice (CD45.1^{+}) and analysed as described above. (D) Quantification of CD45.1^{+} thymocytes or CD45.2^{+} thymocytes as described above in (C) (n=3). (E) Surface staining of thymocytes from irradiated CD45.1^{+}CD45.2^{+}mice, which were reconstituted by mixed Sin1 KO BM cells (CD45.2^{+}) and C57BL/6 CD45.1 mice (CD45.1^{+}) cells, and analysed as described above. (F) Quantification of CD45.1^{+} thymocytes or CD45.2^{+} thymocytes as described above in (E) (n=3). (G) Thymocyte distribution in mice reconstituted by mixed bone marrow (WT (CD45.2^{+}) and C57BL/6 CD45.1 (CD45.1^{+})). Thymocyte subgroups were distinguished by surface mark, and then the ratio of CD45.1^{+} and CD45.2^{+} cells were compared in each group. (H) Thymocyte distribution in mice reconstituted by mixed bone marrow (Sin1KO (CD45.2^{+}) and C57BL/6 CD45.1 (CD45.1^{+})). CD45.1^{+} and CD45.2^{+} cells were compared in each group as (G) Error bars shows mean ± SD. Significance was determined by two-tailed Student's *t* test (*, $p < 0.05$; ns, no significant difference).

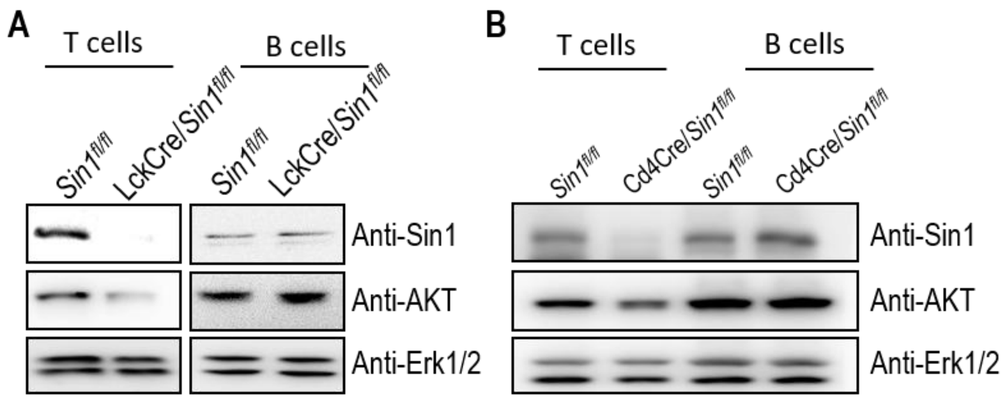

Supplementary Figure S4. Deletion Sin1 in T cells from LckCre/*Sin1*$^{fl/fl}$ and Cd4Cre/*Sin1*$^{fl/fl}$ mice.

(A) Immune blot assay of Sin1 expression in T cells and B cells isolated from splenocytes of *Sin1*$^{fl/fl}$ and LckCre/*Sin1*$^{fl/fl}$ mice. AKT and Erk1/2 expression were served as loading controls. (B) Immune blot assay of Sin1 expression in T cells and B cells isolated from splenocytes of *Sin1*$^{fl/fl}$ and Cd4Cre/*Sin1*$^{fl/fl}$ mice. AKT and Erk1/2 were served as controls.

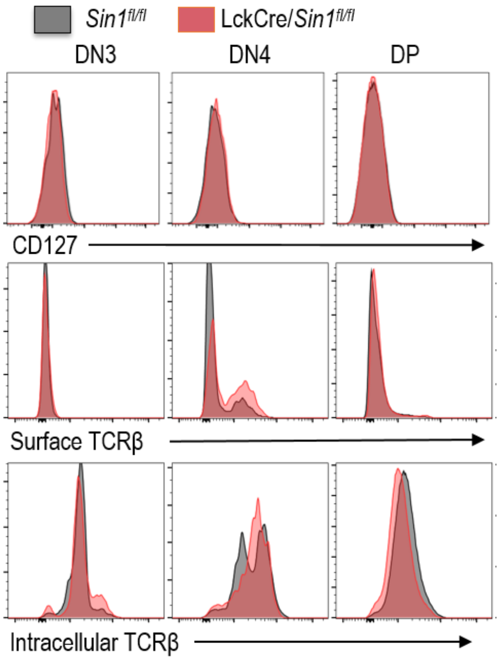

Supplementary Figure S5. Sin1 deficiency does not affect CD127 and TCRβ expression in thymocytes

CD127 and TCRβ expression from freshly isolated thymocytes were determined by FACS staining with a panel of antibodies against CD4, CD8, CD25, CD44, CD127, and TCRβ. Histographies show the surface expression of CD127 and TCRβ, or the intracellular expression of TCRβ gated from $CD4^{-}CD8^{-}$ double negative (DN) stage 3 and DN4, or $CD4^{+}CD8^{+}$ double positive (DP) as indicated.

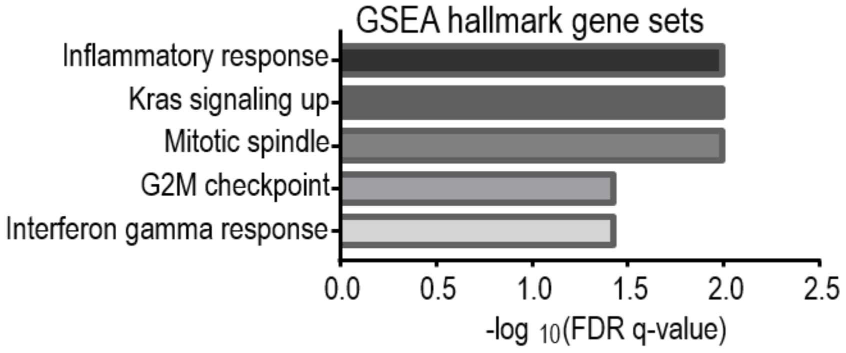

Supplementary Figure S6. Gene sets upregulated in DN thymocytes of Sin1 deficient mice by GSEA analysis

RNA was extracted from DN thymocytes that were sorted from the total thymocytes of the *Sin1*$^{fl/fl}$ and LckCre/*Sin1*$^{fl/fl}$ mice and sequenced on an Illumina Nextseq 500 in a 75 bp paired-end configuration.

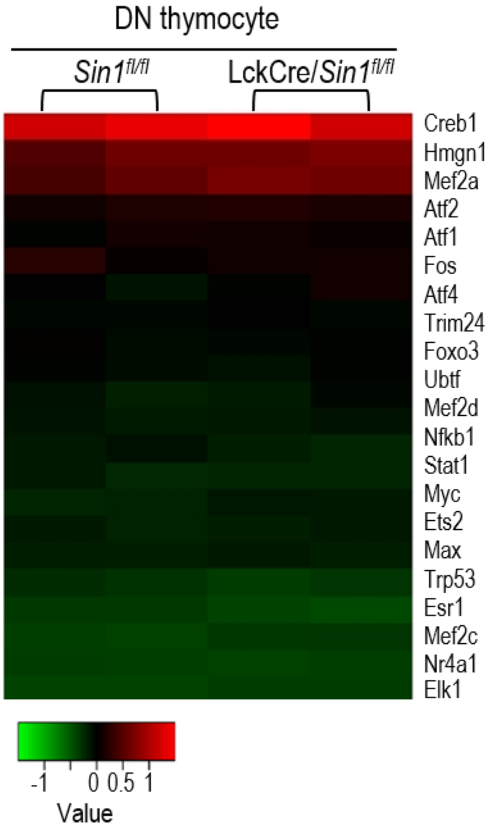

Supplementary Figure S7. Sin1 deficiency does not alter the downstream of MAPK signal pathways

RNA-Seq analysis of gene expression in $CD4^{-}CD8^{-}$ double negative (DN) stage thymocytes. RNA was extracted from purified DN thymocytes of *Sin1*$^{fl/fl}$ or LckCre/*Sin1*$^{fl/fl}$ mice as indicated, and sequenced on an Illumina Nextseq 500 machine in a 75 bp paired-end configuration. Downstream of MAPK signal were selected and analyzed by R package DESeq2.

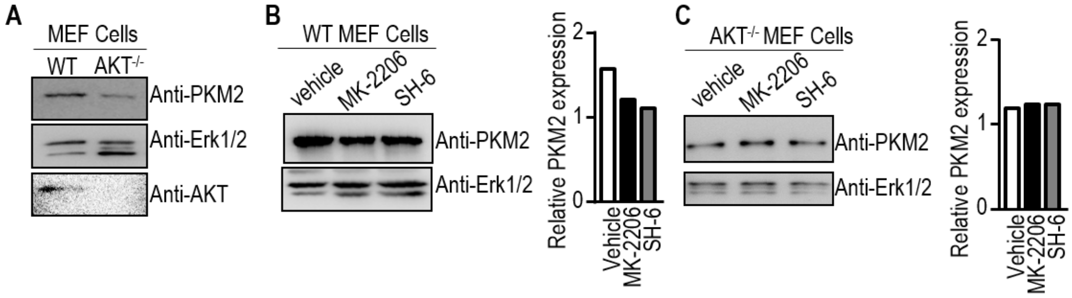

Supplementary Figure S8. AKT controls PKM2 expression in MEF cells.
(A) Immune blot assay for PKM2 expression in WT and AKT1/2$^{-/-}$ MEF cells (AKT$^{-/-}$). Whole cell extracts were prepared for immunoblotting for PKM2 expression, and Erk1/2 is served as a loading control. (B) Immune blot assay for PKM2 expression in WT MEF cells treated with vehicle or 10 μM of MK-2206 or SH-6 overnight. Whole cell extracts were prepared for immunoblotting for PKM2 expression, and Erk1/2 is served as a loading control. The right bar shows the relative PKM2 expression normalized to Erk1/2 level. (C) Immune blot assay for PKM2 expression in AKT1/2$^{-/-}$ MEF cells (AKT$^{-/-}$) treated with vehicle or 10 μM of MK-2206 or SH-6 overnight. Whole cell extracts were prepared for immunoblotting for PKM2 expression, and Erk1/2 is served as a loading control. The right bar shows the relative PKM2 expression normalized to Erk1/2 level.

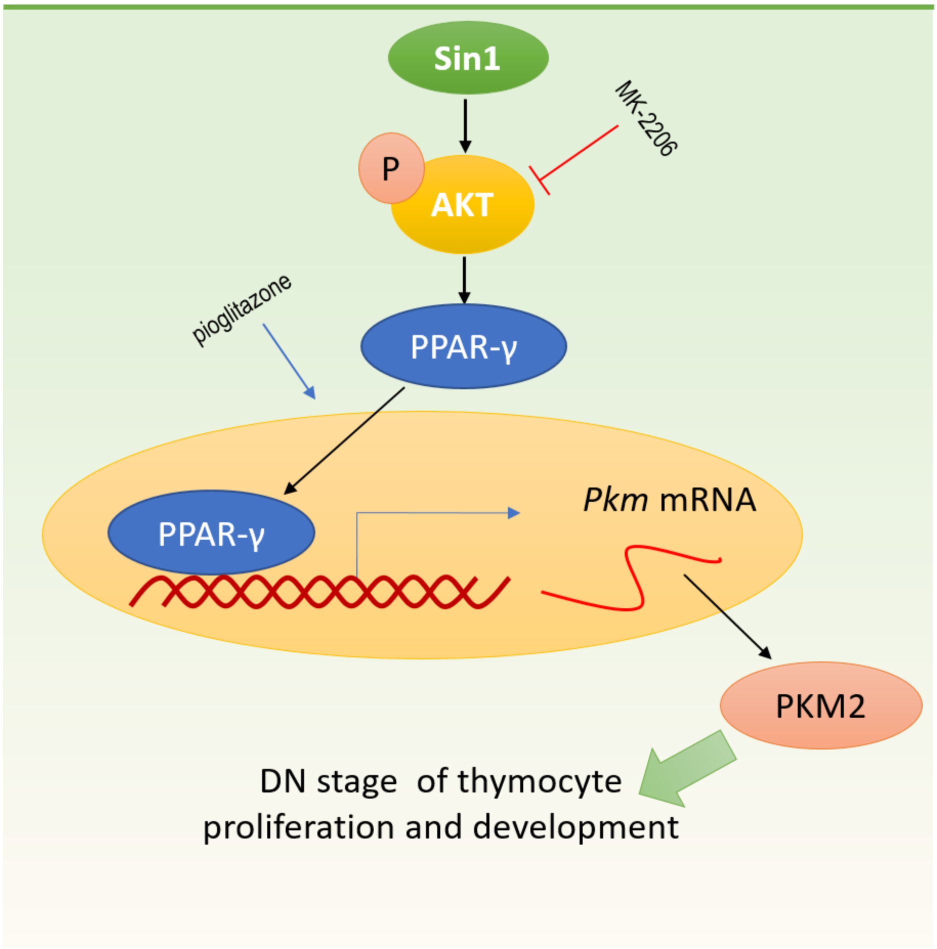

Supplementary Figure S9. proposed model illustrating the Sin1-mediated regulation of early thymocyte proliferation and development

Sin1 first mediates AKT phosphorylation at Thr450 and Ser473, which activates it to phosphorylate PPAR-γ, leading to PPAR-γ nuclear translocation to upregulate PKM2 expression. Augmented PKM2 expression is critical for thymocyte glycolysis and CD4/CD8 double negative thymocyte proliferation and development.

II. Supplementary tables

Supplementary Table S1. Primers used for genotyping.

Primers	Sequence
P1	5'-TGTATTAGCCTGCACAAGGAG T-3'
P2	5'-CAACGGGTTCTTCTGTTAGTCC-3'
P3	5'-ATGGACTGCTTGCCTGAACT-3'
P4	5'-ATTGAACTGATGGCGAGCTC-3'

Supplementary Table S2. Primers used for Real time-PCR

Primers	Sequence
pkm2 for	5’-TCGCATGCAGCACCTGATT-3’
pkm2 rev	5’-CCTCGAATAGCTGCAAGTGGTA-3’
PKM1 for	5’-GCTGTTTGAAGAGCTTGTGC-3’
PKM1 rev	5’-TTATAAGAGGCCTCCACGCT-3’

Supplementary Table S3. Transcriptions significantly more abundant in Sin1 deficient DN thymocytes

Gene	FC	P-value	Gene	FC	P-value
AA465934	1.55314	0.001417	Gm14085	1.608389	0.013904
Il5ra	1.363755	0.002093	9230116N13Rik	1.370019	0.01408
2610020C07Rik	1.303183	0.003306	Gm20939	1.32094	0.015825
C030037D09Rik	1.820512	0.004424	Papss2	1.304995	0.016756
Slc6a19	1.518907	0.005496	Akr1c12	1.450124	0.018073
Mnd1-ps	1.440158	0.005885	1110019D14Rik	1.321578	0.018112
Pou4f1	1.318267	0.006857	Myof	1.308711	0.018177
Plscr4	1.314385	0.007784	Bambi-ps1	1.347448	0.020048
Tlcd2	1.364191	0.008101	Gm43549	1.56196	0.020489
Gm5511	1.379382	0.008436	Necab1	1.524796	0.020882
Shc4	1.320668	0.009011	2310009B15Rik	1.302137	0.021366
5830405F06Rik	1.355672	0.009949	Iigp1	1.533071	0.024651
Arl6	1.31049	0.00995	1700097N02Rik	1.373809	0.02518
Gm6245	1.360426	0.010015	Gm13577	1.314502	0.030514
Neb	1.339836	0.011115	Prtg	1.317325	0.033327
Xaf1	1.489296	0.01191	9630013D21Rik	1.413191	0.037501
Adam11	1.620735	0.01217	6820402A03Rik	1.393233	0.03925
A430035B10Rik	1.304762	0.012603	Fgfbp3	1.322822	0.04149
Gm13051	1.402711	0.013674	Gm2885	1.485788	0.044029
Mnd1	1.33225	0.013881	Gm12185	1.434093	0.049829

FC, fold change. P-value is determined by negative test.

Supplementary Table S4. Transcriptions significantly less abundant in Sin1 deficient DN thymocytes

Gene	FC	P-value	Gene	FC	P-value
S100a9	0.161502	0.001533	Gm8399	0.642312	0.005129
Ngp	0.169441	0.002351	Gm14176	0.64235	0.007019
Chil3	0.226392	0.000139	Slc25a22	0.642639	0.010493
S100a8	0.232356	0.000205	Gm7901	0.646091	0.004392
Bckdha	0.362002	0.008423	Tigar	0.646311	0.009352
Cd74	0.378199	0.001413	Chst3	0.646559	0.008718
Cd7	0.379179	0.006605	Ighd	0.646751	0.007029
Fcmr	0.391279	0.005821	Gm6472	0.647048	0.005777
Lyz2	0.403436	0.000317	Arrb1	0.647771	0.006609
Ctsw	0.414476	0.00752	Tln1	0.647883	0.004582
Iglc3	0.430159	0.0065	Sgta	0.648246	0.009471
Gm15564	0.448766	0.00389	Dennd5b	0.648501	0.007614
Gm8730	0.450076	0.012437	Rpl9-ps6	0.648858	0.008506
Gzmb	0.451077	0.010581	Slc38a10	0.649196	0.001817
Rin3	0.466499	0.006815	Rpl36a-ps3	0.64935	0.009398
Cd19	0.470244	0.012427	Gpsm3	0.649791	0.007871
H2-Aa	0.474232	0.003865	Amica1	0.650198	0.012462
Hsf1	0.476609	0.007287	Slc25a45	0.651636	0.011236
Dock2	0.482097	0.005092	Shisa5	0.652149	0.005779
Il2rb	0.483449	0.007746	Rps19-ps6	0.653607	0.009071
Itgb7	0.48775	0.008112	Rbck1	0.655172	0.003789
Rpl8	0.488199	0.009002	Rps5	0.655461	0.000185
Pdk2	0.489229	0.004695	Ucp2	0.656961	0.010161
Oaz1-ps	0.498731	0.008059	Tecr	0.657162	0.01423
Gm42418	0.503181	0.010602	Gm1840	0.657203	0.0101
H2-Ab1	0.513372	0.003371	Plekho2	0.65777	0.010597
Slc25a10	0.51487	0.010811	B4galt5	0.660613	0.004866
Capg	0.515422	0.009359	Bank1	0.661061	0.013856
Myo1f	0.518331	0.01394	Map7d1	0.661613	0.009292
Nbeal2	0.522226	0.003942	Pfdn5	0.661923	0.005227
Eno1	0.524555	0.014605	Syvn1	0.662761	0.006164
Cybb	0.529451	0.010933	Pkib	0.6631	0.008575
Card11	0.529874	0.007035	Cyth4	0.664992	0.006469
Lilrb4a	0.531902	0.00187	Hvcn1	0.665405	0.01341
B4galnt1	0.532433	0.005082	Cyc1	0.665999	0.006515
Gm4737	0.538116	0.007638	Gm15427	0.667235	0.008337
Epn1	0.544527	0.004539	2310067B10Rik	0.668588	0.008127
Blnk	0.548162	0.001092	Pfn1	0.669565	0.001157
Cd72	0.54884	0.000553	Parvg	0.670119	0.009968

Gene	FC	P-value	Gene	FC	P-value
Ncf1	0.552351	0.012562	Fmnl1	0.670425	0.00264
Anxa1	0.552807	0.011799	Pnpla2	0.670804	0.004846
Gm6560	0.55563	0.011439	Scd1	0.671904	0.005082
Cd180	0.55735	0.002378	Map4k1	0.672962	0.007757
Pax5	0.55832	0.005576	Tpi1	0.673148	0.000648
Lars2	0.559982	0.00734	Atad3a	0.673453	0.011443
Cr2	0.561151	0.001993	Gpsm1	0.676724	0.004376
Pold2	0.561959	0.003203	Eef1a1	0.677608	0.004754
Cd83	0.562	0.010713	Tmc8	0.677807	0.001455
Cd52	0.562269	0.013828	Pkm	0.678618	0.012051
F2r	0.562324	0.008896	Pde1b	0.679204	0.000238
Peg13	0.562871	0.013196	Lcp1	0.680271	0.000997
Abca2	0.563707	0.003454	Actb	0.680423	0.003308
Sh3bgrl3	0.563956	0.006736	Mapk3	0.68125	4.14E-05
Pstpip1	0.567305	0.00427	Creb3	0.682085	0.010651
Lsp1	0.568381	0.005286	Gm5526	0.682618	0.010091
Plcg2	0.58714	0.008432	Rpl3-ps1	0.682984	0.007465
Actg1	0.588768	0.008388	Ftl1	0.684414	0.00855
Zfp551	0.59207	0.001268	Traf7	0.685609	0.006467
Rasgrp3	0.593172	0.012222	Ehmt2	0.686055	0.00284
Atp5l	0.5947	0.000679	Coro1a	0.686408	0.004692
Ptpn6	0.59615	0.003034	43352	0.686797	0.010607
Atp13a2	0.596203	0.008671	Mybbp1a	0.68689	0.001123
Guk1	0.598342	0.010388	Hmgxb3	0.688444	0.008484
Fermt3	0.59864	0.011264	St6gal1	0.688531	0.010072
Aldoa	0.599694	0.001065	Tkt	0.689794	0.002042
Myo1g	0.600162	0.004535	Rps11-ps1	0.690878	0.001655
Eif3k	0.600426	0.011289	Gm5428	0.690895	0.005982
Dgkz	0.600679	0.002683	Gnl1	0.691202	0.005992
Eef2	0.602145	0.000706	Anxa6	0.691265	0.006899
Pfkl	0.603828	0.010109	Ubr4	0.691413	0.003168
Flna	0.604609	0.008229	Gm7536	0.692604	0.005645
Unc13d	0.605433	0.007234	Cluh	0.692859	0.005581
Zyx	0.606438	0.010563	Acadvl	0.695137	0.011243
Arhgef1	0.608366	0.008638	Helz2	0.695361	0.000119
Unc45a	0.612875	0.000277	Gm10288	0.695496	0.003554
H3f3c	0.61775	0.011055	Gnb2l1	0.696193	0.004499

Gene	FC	P-value	Gene	FC	P-value
Gm15772	0.620428	0.00247	Rpl41	0.696705	0.010824
Sorl1	0.623466	0.011337	Ech1	0.696769	0.004377
Kri1	0.623579	0.000847	Zgpat	0.697692	0.002003
Tmem222	0.624288	0.003672	Gm4366	0.698247	0.00607
Srm	0.625456	0.006284	Cuedc2	0.70106	0.007913
Sipa1	0.628109	0.006332	Aurkaip1	0.701097	0.011578
Lgals1	0.631843	0.011414	Armc7	0.701478	0.012745
Gm13456	0.633046	0.006196	Smarca4	0.701896	0.010451
Fcrla	0.633765	0.007981	Itgal	0.702346	0.00812
Lrrk2	0.633827	0.012972	Cst3	0.702962	0.000985
Mast3	0.635784	0.008608	Uqcrh	0.703023	0.000225
Cdc37	0.637349	0.000258	Mapkap1	0.707385	0.017093
Akap8l	0.639241	0.013949	Gpi1	0.709376	6.55E-15
Gm7665	0.639634	0.012652	Gpx4	0.710535	0.001771
Sbf1	0.640244	0.013882	Idh3g	0.729158	0.001546
Trappc9	0.640567	0.002821	Uqcrc1	0.763413	0.009118
Gm1821	0.64079	0.003399	Ndufs6	0.77162	0.008609
Lrp5	0.642178	0.007606	Mdh2	0.798854	0.004548
Rpl9-ps7	0.642228	0.00229	Ndufv3	0.840939	0.014215

FC, fold change. P-value is determined by negative test.
